# Increased duplex stabilization in porphyrin-LNA zipper arrays with structure dependent exciton coupling†
†Electronic supplementary information (ESI) available: Synthetic procedures for the building blocks and DNA strands, full spectroscopic analysis of the ssDNA and duplex systems. See DOI: 10.1039/c5ob01681a



**DOI:** 10.1039/c5ob01681a

**Published:** 2015-09-29

**Authors:** Daniel G. Singleton, Rohanah Hussain, Giuliano Siligardi, Pawan Kumar, Patrick J. Hrdlicka, Nina Berova, Eugen Stulz

**Affiliations:** a School of Chemistry and Institute for Life Sciences , University of Southampton , Highfield , Southampton , SO17 1BJ , UK . Email: est@soton.ac.uk ; http://www.southampton.ac.uk/chemistry/about/staff/est.page?; b Diamond Light Source , Harwell Science and Innovation Campus , Didcot , Oxfordshire OX11 0DE , UK; c Department of Chemistry , University of Idaho , Moscow , ID 83844 , USA; d Department of Chemistry , Columbia University , 3000 Broadway , New York , NY 10027 , USA

## Abstract

Porphyrins were attached to LNA uridine building blocks *via* rigid 5-acetylene or more flexible propargyl-amide linkers and incorporated into DNA strands. The systems show a greatly increased thermodynamic stability when using as little as three porphyrins in a zipper arrangement. Thermodynamic analysis reveals clustering of the strands into more ordered duplexes with both greater negative ΔΔ*S* and ΔΔ*H* values, and less ordered duplexes with small positive ΔΔ*S* differences, depending on the combination of linkers used. The exciton coupling between the porphyrins is dependent on the flanking DNA sequence in the single stranded form, and on the nature of the linker between the nucleobase and the porphyrin in the double stranded form; it is, however, also strongly influenced by intermolecular interactions. This system is suitable for the formation of stable helical chromophore arrays with sequence and structure dependent exciton coupling.

## Abstract 2

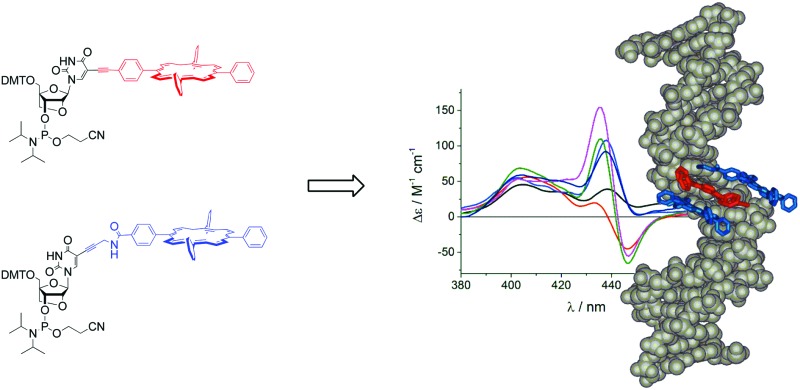
LNA-porphyrin building blocks were used to create stable zipper-porphyrin DNA arrays, which were analysed in detail with CD spectroscopy and thermodynamic studies.

## Introduction

DNA has become very attractive as a supramolecular scaffold in nano-biotechnology,^1–7^ because it offers the possibility to add functionality exceeding its role in biological contexts.^8^ Chemical modification of nucleotides at either the nucleobase or sugar moiety with aromatic compounds or metal complexes,^9–13^ or replacement of the nucleobase^14–16^ or the nucleoside altogether,^17,18^ allows for attachment of a huge variety of functional substituents,^19^ in particular for energy or electron transfer^20–23^ or sensing applications.^24,25^ In this way, helical chromophore arrays were generated to create photonic wires or mimic the antenna effect of the photosynthetic system, either through standard DNA synthesis or *via* enzymatic DNA extension.

Building blocks consisting of porphyrins covalently attached to nucleotides^26–28^ offer great versatility due to their tuneable electrochemical and optical properties. Methods for attachment include modification of the nucleobases,^29–35^ ribofuranose residues,^35–43^ phosphate backbone^44–47^ and using acyclic linkers.^18,48–51^ This led to assemblies in which porphyrin residues were positioned as 3′- or 5′-molecular caps,^39,48,50,52,53^ and used instead of a nucleobase in the middle of the helix^18,49,51^ or as a label in the minor^37,42,43,47^ and major^30–35^ grooves. The systems have been used to detect structural switching using chiroptical methods,^39,46,54,55^ realize viral DNA sensing using micro-electrochemistry,^56^ and form reversible photonic wires through hybridization.^33^ Porphyrins have been shown to be particularly useful substituents for DNA based bio-nanotechnology, allowing the formation of DNA tubes^57^ or acting as lipophilic anchors for insertion of nanopores^58,59^ and electronic systems into lipid bilayers.^60^

Incorporation of multiple porphyrins can lead to substantial thermodynamic destabilization of the duplex when only one strand is modified;^30^ this can be compensated by arranging the porphyrins in interstrand zippers, resulting in the formation of very stable duplexes.^31,33^ Here we investigated the use of porphyrin-LNA (LNA = locked nucleic acid, LNA-P) as building blocks due to its potential ability to further stabilize the DNA duplex.^61^

The advantage of using C5-functionalization of pyrimidine bases is that the substituents can precisely be oriented into the major groove of the DNA.^30,62^ In this respect, the LNA modifier is identical to the 2′-deoxyuridines (dU) normally used for modifying DNA. This has been shown for other systems using C5-modified LNA,^63–65^ and generally contrasts the attachment of substituents on the 2′-position of the ribose which positions them into the minor groove.^62^ Here we show that the LNA modification indeed has a positive effect on duplex stability despite the very large and hydrophobic porphyrin substituents, and that the excitonic coupling between the chromophores can be modulated by selecting different linkers to the substituent.

## Results and discussion

### Synthesis of the LNA building blocks

**Scheme 1:**
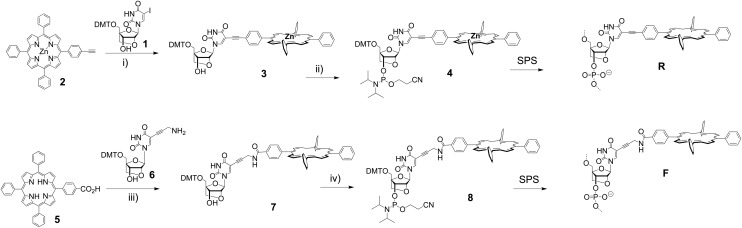
Synthesis of the building blocks and DNA. Reagents and conditions: (i) Pd(PPh_3_)_4_, CuI, TEA, DMF, 91%; (ii) CEP-Cl, DIPEA, DCM, quant.; (iii) EDC, HOBt, DMAP, DCM, 68%; (iv) CEP-Cl, DIPEA, DCM, quant. SPS: Solid Phase Synthesis of DNA.

We have synthesized two porphyrin-LNA building blocks, where the porphyrin is attached either *via* a short rigid acetylene linker (**4**), or *via* a more flexible propargyl-amide linker (**8**); the synthetic route is outlined in Scheme 1. The 5-iodo-LNA-U building block **1** was obtained by methods previously reported.^65^ Attachment of acetylene porphyrin **2** ^30^ was realized using Sonogashira coupling to give **3** in 91% yield. The carboxylate porphyrin **5** ^31^ was coupled to the propargyl-amine-LNA-U **6** ^63^ using EDC/HOBt mediated peptide coupling giving access to **7** in 68% yield. The building blocks were transformed into their phosphoramidite counterparts and used directly for solid phase synthesis of the oligodeoxynucleotide (ODN) strands (see ESI† for details of the synthesis and Table 1 for sequences). Incorporation of **4** gave the **R**-series, whereas **8** gave the **F**-series denoting rigid and flexible linker, respectively, and the unmodified ODN sequence is denoted as **U**-series. The coupling of the modified building blocks generally was highly efficient according to the trityl monitoring using an extended 6 min coupling protocol; the isolated yields after HPLC purification were between 23% and 70% according to UV-measurements (see ESI†). The central zinc metal in **4**, which is inserted to prevent copper metallation during the Sonogashira coupling, is lost during SPS due to the acidic conditions in DMT removal, thus both modifiers **R** and **F** in the DNA are obtained as free base porphyrins. The strands were analysed by UV-vis and CD spectroscopy, and by thermal denaturation experiments to obtain insight into both thermodynamic and optical aspects of the LNA arrays.

**Table 1:** Thermodynamic parameters of porphyrin arrays formed from homo- or hetero-duplex systems^*a*^

	ODN duplex	*T*_m_/°C [Δ*T*_m_]	Δ*H*/kJ mol^–1^ [ΔΔ*H*]	–*T* ^298^Δ*S*/kJ mol^–1^ [Δ(–*T* ^298^Δ*S*)]	Δ*G* ^298^/kJ mol^–1^ [ΔΔ*G* ^298^]
a	U1 5′-GTG ATA TGC	37.6	–279 ± 0.2	233	–46.4
	U2 3′-CAC TAT ACG	39.7^*b*^	–285 ± 12^*b*^	239^*b*^	–45.9^*b*^
b	R1 5′-GTG A**R**A TGC	32.5 [–5.1]	–261 ± 1 [+18]	220 [–13]	–40.7 [+5.7]
	U2 3′-CAC TAT ACG				
c	U1 5′-GTG ATA TGC	33.0 [–4.6]	–281 ± 0.6 [–2]	239 [+6]	–41.9 [+4.5]
	R2 3′-CAC **R**AT ACG	34.5^*b*^ [–5.2]	–283 ± 6^*b*^ [+2]	241 [+2]	–42.1^*b*^ [+4.3]
d	U1 5′-GTG ATA TGC	31.4 [–6.2]	–255 ± 0.7 [+24]	215 [–18]	–40.3 [+6.1]
	R3 3′-CAC TA**R** ACG				
e	U1 5′-GTG ATA TGC	27.7 [–9.9]	–254 ± 0.5 [+25]	217 [–16]	–36.6 [+9.8]
	R4 3′-CAC **R**A**R** ACG				
f	F1 5′-GTG A**F**A TGC	36.7 [–0.9]	–356 ± 0.2 [–77]	308 [+75]	–47.7 [–1.3]
	U2 3′-CAC TAT ACG				
g	U1 5′-GTG ATA TGC	35.7 [–1.9]	–257 ± 0.3 [+22]	214 [–19]	–42.6 [+3.8]
	F2 3′-CAC **F**AT ACG				
h	U1 5′-GTG ATA TGC	35.4 [–2.2]	–333 ± 0.1 [–54]	287 [+54]	–45.9 [+0.5]
	F3 3′-CAC TA**F** ACG				
i	U1 5′-GTG ATA TGC	29.9 [–7.7]	–203 ± 0.7 [+76]	165 [–68]	–37.5 [+8.9]
	F4 3′-CAC **F**A**F** ACG	28.9^*b*^ [–10.8]	–222 ± 7^*b*^ [+63]	186^*b*^ [–53]	–35.7^*b*^ [+10.7]
j	R1 5′-GTG A**R**A TGC	35.6 [–2.0]	–357 ± 0.8 [–78]	310 [+77]	–47.2 [–0.8]
	R2 3′-CAC **R**AT ACG				
k	R1 5′-GTG A**R**A TGC	35.5 [–2.1]	–368 ± 0.2 [–89]	321 [+88]	–46.9 [–0.5]
	R3 3′-CAC TA**R** ACG				
l	R1 5′-GTG A**R**A TGC	39.7 [+2.1]	–362 ± 0.2 [–83]	312 [+79]	–50.4 [–4.0]
	R4 3′-CAC **R**A**R** ACG				
m	F1 5′-GTG A**F**A TGC	36.8 [–0.8]	–344 ± 0.2 [–65]	297 [+64]	–46.7 [–0.3]
	F2 3′-CAC **F**AT ACG				
n	F1 5′-GTG A**F**A TGC	35.9 [–1.7]	–256 ± 0.3 [+23]	213 [–20]	–42.5 [+3.9]
	F3 3′-CAC TA**F** ACG				
o	F1 5′-GTG A**F**A TGC	40.9 [+3.3]	–274 ± 0.2 [+5]	226 [–7]	–48.3 [–1.9]
	F4 3′-CAC **F**A**F** ACG				
p	R1 5′-GTG A**R**A TGC	37.7 [+0.1]	–344 ± 0.2 [–65]	295 [+62]	–48.5 [–2.1]
	F2 3′-CAC **F**AT ACG				
q	R1 5′-GTG A**R**A TGC	37.8 [+0.2]	–270 ± 0.2 [+9]	225 [–8]	–45.5 [+0.9]
	F3 3′-CAC TA**F** ACG	38.2^*b*^ [–1.5]	–348 ± 4^*b*^ [–63]	300b [+61]	–47.8^*b*^ [+1.4]
r	R1 5′-GTG A**R**A TGC	41.3 [+3.7]	–349 ± 0.1 [–70]	297 [+70]	–52.0 [–5.6]
	F4 3′-CAC **F**A**F** ACG				
s	F1 5′-GTG A**F**A TGC	38.0 [+0.4]	–280 ± 0.5 [–1]	234 [+1]	–46.3 [+0.1]
	R2 3′-CAC **R**AT ACG				
t	F1 5′-GTG A**F**A TGC	40.5 [+2.9]	–269 ± 0.4 [+10]	221 [–12]	–47.8 [–1.4]
	R3 3′-CAC TA**R** ACG				
u	F1 5′-GTG A**F**A TGC	42.6 [+5.0]	–287 ± 0.4 [–8]	237 [–4]	–50.3 [–3.9]
	R4 3′-CAC **R**A**R** ACG				

^*a*^Data obtained by thermal denaturing using UV monitoring.

^*b*^Data obtained by thermal denaturing using CD monitoring; Δ*T*_m_ is calculated relative to the base value obtained by the same method. The *T*_m_ values were obtained from the first derivative of the melting curves at 260 nm (0.1 °C min^–1^, no hysteresis was observed; 2.5 μM DNA, 50 mM phosphate buffer, 100 mM NaCl, 1 mM Na_2_EDTA, pH 7.0).

DNA duplexes were designed to feature either one porphyrin at a central position (Table 1, entries b, c, d, f, g, h), two porphyrins on one of the strands (entries e, i), interstrand zipper arrays with two porphyrins (entries j, k, m, n, p, q, s, t), or three porphyrins (entries l, o, r, u).

### Thermodynamic stability

The DNA duplexes, which have the LNA-porphyrin building block incorporated in only one strand, generally show a decrease in thermal stability (b–i). This is more pronounced for the rigid acetylene linker (**R**-series) than for the propargyl-amide linker (**F**-series). Clearly, the attachment of the porphyrin outweighs the stabilizing effect of the LNA skeleton, which is in the range of +4.0–+6.5 °C in this sequence context.^65^ Note that we define “sequence context” as placing the porphyrins at different positions within the given DNA sequence, resulting in variation of the porphyrin environment with respect to the neighbouring DNA sequence. The attachment of two porphyrins in complementary strands using the same linker (j, k, m, n) equally does not show duplex stabilization. However, forming a two-porphyrin array with different linkers on the complementary strands (p, q, s, t) leads to a compensation of the destabilizing effect, and the duplex **F1**·**R3** actually displays a stabilization of Δ*T*_m_ = +2.9 °C, and with it revealing sensitivity of the system with respect to the linker moiety used. Introduction of three porphyrin-functionalized LNA monomers into DNA duplexes (l, o, r, u) results in substantial thermodynamic stabilization, as revealed by the higher Δ*T*_m_ and lower ΔΔ*G* values; a more detailed discussion on the entropic and enthalpic contributions is given further below.

Detailed analysis of the *T*_m_-values shows that the influence in the zipper-systems is not a simple sum of the modifications. To illustrate, **R1**·**U2** and **U1**·R2 show Δ*T*_m_ = –5.1 °C and Δ*T*_m_ = –4.6 °C, respectively, whereas the duplex **R1**·**R2** shows Δ*T*_m_ = –2.0 °C. The same is the case for **R1**·**R3**, and this effect is even more pronounced in **R1**·**R4**. In contrast, the stabilizing effect is less pronounced when both strands are modified by the flexible linker (Table 1, m–o), suggesting that flexibility is detrimental for array stability. This means that placement of the **R** monomers in zipper arrangements counteracts the inherent destabilizing properties of the **R** monomers more efficiently than is the case with the **F** monomers; in other words, ΔΔ*T*_m_ is larger for **R** than for **F** when comparing **U**·**X** to **X**·**X**. That being said, porphyrin zippers that entail **F** monomers, and especially mixtures of **F** and **R** monomers, are the most stable in absolute terms. **F1**·**R4** shows the highest stabilization effect with a Δ*T*_m_ = +5.0 °C. This increase of 1.7 °C per porphyrin is decisively larger than that seen with the dU-P system (0.3–0.5 °C),^31,33^ suggesting that array formation between porphyrin units is more stabilising in LNA-P based systems.

In the closely related dU-zipper system, which contains six porphyrins per modified strand, the order of stabilisation was found to be **U**·**F** > **F**·**F** > **U**·**R** > **R**·**F** > **R**·**R**.^31^ In the LNA-system, the order is **F**·**R** > **F**·**F** > **R**·**R** > **U**·**F** > **U**·**R**. This suggests that (i) the less restricted dU-P is better tolerated by an unmodified complementary strand, and in both dU-P and LNA-P the flexible linker is beneficial; (ii) a certain degree of flexibility is necessary in order to form stable zipper arrays; (iii) the preorganised LNA modifier forms a duplex in which the mixed porphyrin array forms a better stack whereas dU adjusts better to flexible linker. It was suggested previously through molecular modelling that the mixed porphyrin system forms a more evenly distributed porphyrin arrangement in the major groove of the duplex.^31^ This, however, applies only to the zipper-arrangement; the thermodynamic analysis of all combinations is more complex as discussed further below. An influence of the linker length in **F** cannot be ruled out to act in combination with the increased flexibility.

Overall, hydrophobic interactions exert a positive effect with increasing number of porphyrins, and the system is very sensitive to the nature of the linker and sequence context of the modification. The melting temperatures were, in a few cases, also determined using CD-melting (Table 1) and are in good agreement with the UV-melting temperatures. The overall duplex structure continues to exhibit the B-form helical conformation where the melting is reflected by a global change of the molecule, and is not affected by secondary structures or intermolecular interactions induced by the porphyrin (see below).

**Fig. 1:**
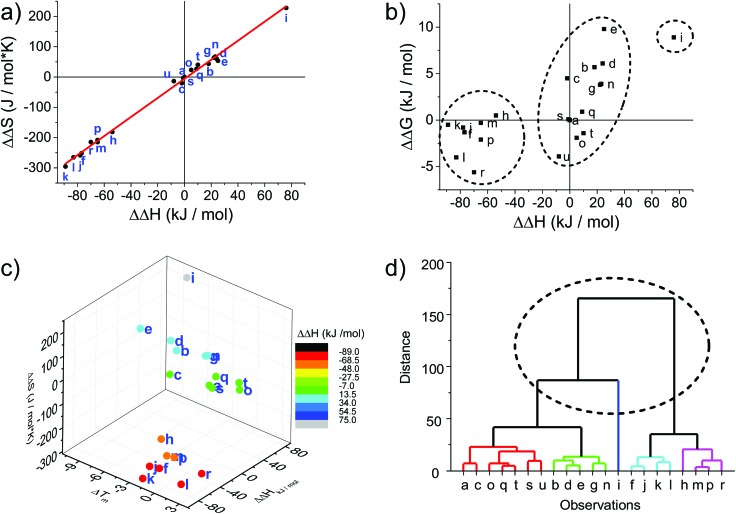
Thermodynamic plots of the porphyrin-LNA duplexes. Data are taken from Table 1. (a) Enthalpy–entropy compensation with linear regression [ΔΔ*S* = –5.88 + (3.15 K)ΔΔ*H*; **R2** = 0.996]. (b) Correlation between ΔΔ*H* and ΔΔ*G* ^298^ demonstrating that there is a linear correlation between ΔΔ*G* and ΔΔ*H*.^66^ (c) Enthalpy–entropy-change in Δ*T*_m_ correlation showing further clustering when compared to net change of entropy. (d) Hierarchical analysis using the euclidean distance carried out by OriginLab software presenting five clusters of duplex samples. The dashed circle indicates the classification of the three groups identified in (a), (b) and (c); the main groups can be further divided into five major subgroups.

The melting curves were fitted to provide thermodynamic parameters (see Table 1 and ESI†). The differences in enthalpy and entropy, as expressed in ΔΔ*H* and ΔΔ*S*, are illustrative of the influence of the modification compared to the unmodified DNA duplex. Plotting ΔΔ*S vs.* ΔΔ*H* shows a linear correlation with an intercept very close to the origin (red line in Fig. 1a); the intercept of –5.8 J mol^–1^ and slope of 3.15 K agrees well with the reported values for a series of LNA strands (intercept 9.5 J mol^–1^, slope 2.95 K).^66^

From a global point of view, the plots of thermodynamic parameters ΔΔ*S vs.* ΔΔ*H* revealed one isolated and two main groups of duplex architecture systems (Fig. 1a). The group comprising the f, h, j, k, l, m, p and r duplex systems with both greater negative ΔΔ*S* and ΔΔ*H* values should be considered to be more ordered compared to the reference duplex “a” relative to which the ΔΔ*S* and ΔΔ*H* values were calculated. The second group comprising the remaining duplex systems (except “i”) with small positive ΔΔ*S* differences can be viewed as less ordered than “a”. The duplex “i” (**U1**·**F4**) clearly falls outside the clustering, indicating that the use of two flexible **F** monomers on one DNA strand is enthalpically very unfavourable. The systems **U**·**F**, **R**·**R** and **R**·**F** fall mainly into the first group, and the systems **U**·**R**, **F**·**F** and **F**·**R** can be predominantly found in the second clustering.

The enthalpy–entropy compensation has been discussed for LNA, and since the plot of ΔΔ*G* ^298^ *vs*. ΔΔ*H* shows a positive correlation rather than random scattering (Fig. 1b) it suggests that the compensation is not due to experimental error, and that the porphyrin-LNA building blocks can stabilize the duplex by either preorganization or improved stacking, but not both simultaneously.^66^

From the ΔΔ*G* ^298^ and ΔΔ*H* plot (Fig. 1b), it can be deduced that the duplex architecture of the first group is energetically more favourable than that of the reference duplex “a”, whereas for the second group the difference is smaller and towards less stable duplex formation than “a” with “i” forming the least stable duplex. The 3D plot of *T*_m_, ΔΔ*H* and ΔΔ*S* is a better way of representing the thermodynamic parameters (Fig. 1c) and shows the trends of the three groups in a more accurate and complete way. The cluster analysis as shown in Fig. 1d is consistent with the broad classification of the duplex architectures as revealed in Fig. 1a–c. In fact, the systems can be represented in five major subgroups with the systems {a, c, o, q, s, t, u} with mainly **F**·**R** zippers; {b, d, e, g, n} with mainly **U**·**R** duplexes; {i}; {f, j, k, l} with mainly **R**·**R** zippers; and {h, m, p, r} with mainly **R**·**F** zippers. Outliers are present in each of the groups.

The destabilizing characteristics of **R**-modified ODNs (**R**·**U** or **U**·**R**) are of an enthalpic nature (ΔΔ*H* > 0 kJ mol^–1^), consistent with structural perturbation by the porphyrin in the major groove. The underlying structural reasons for the destabilizing properties of the monomer **F** (**F**·**U** or **U**·**F**) appear to be much more sequence/position-dependent since highly unfavourable entropic contributions are observed in some cases (see Δ(–*T* ^298^Δ*S*) for **F1**·**U2**), while strongly unfavourable enthalpic contributions are seen in others (see ΔΔ*H* for **U1**·**F2**). In the zipper arrays, **R** stabilizes the duplexes through enthalpic contributions, in agreement with the formation of π–π stacks (ΔΔ*H* ≪ 0 kJ mol^–1^ for **R**·**R**). The more flexible **F** in the **F**·**F** arrays stabilizes DNA duplexes through favourable enthalpic contributions in some constructs (–1 zipper constructs) and entropic contribution in others (+1 zipper construct). Finally, the **R**·**F** hetero-arrays are either stabilized due to strong enthalpic contributions (*e.g.*, **R1**·**F2**), or through minor differences in enthalpy/entropy contributions (**F1**·**R3**).

### Circular dichroism of free-base LNA strands

**Fig. 2:**
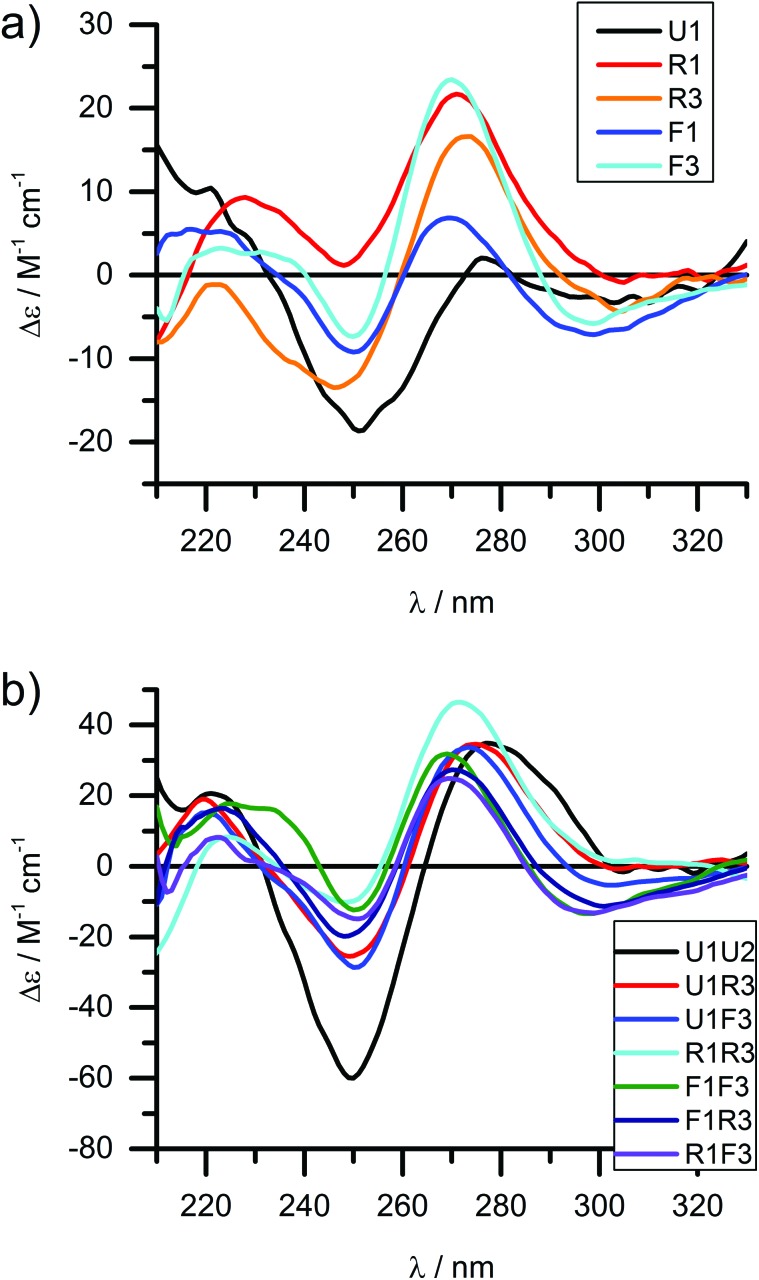
CD spectra of the DNA region of LNA-porphyrin modified ODNs (selected representative examples) in (a) single stranded and (b) double stranded form. 4 μM DNA, 50 mM phosphate buffer, 100 mM NaCl, 1 mM Na_2_EDTA, pH 7.0.

**Fig. 3:**
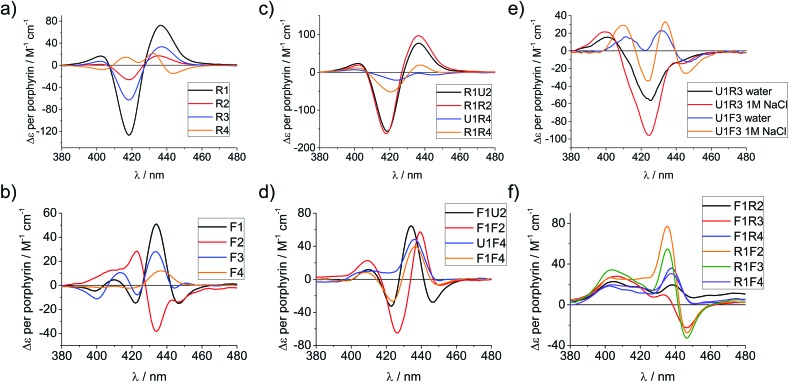
CD spectra of porphyrin-LNA strands of selected examples showing the porphyrin region of the spectra. (a) and (b) spectra of single strands with **LNA-1** or **LNA-2** modification, respectively. (c) and (d) spectra for selected duplexes where the same modifier has been used in both strands. (e) Comparison of stands **U1**·**R3**/**F3** in pure water or in 1 M NaCl (50 mM phosphate buffer) solution. (f) Mixed LNA duplexes where the complementary strands have been modified with different modifiers. Δ*ε* values are given per porphyrin (normalized to the number of porphyrins present). Conditions as in Fig. 2.

Circular dichroism (CD) spectroscopy is a very versatile tool in assessing structural aspects through electronic interactions,^67^ and both the DNA and the porphyrin parts of the spectrum are indicative of the impact of the modifications on structure. Single-stranded LNA-modified ODNs show a signature reminiscent of a DNA·RNA hybrid duplex structure^68,69^ with varying effects that are depending on the sequence context; Fig. 2a displays representative examples for both building blocks (see ESI† for full analysis). This effect is not pronounced in the double stranded ODNs where all systems largely display the structural characteristics consistent of a B-type DNA duplex (Fig. 2b), although the +275 nm band is shifted to lower value, and the Δ*ε* values are reduced, indicating reduced helicity in the modified systems. The interpretation of the porphyrin region of the CD spectra is more complex as it shows large variance with respect to the nature of the modifier, sequences and hybridization state (see Fig. 3 for examples and ESI† for full spectral analysis). In the single-modified porphyrin strands (**R1**–**R3**, **F1**–**F3**), the signatures are more complex than would be anticipated, as either a single positive or single negative Cotton effect should be expected. The bisignate or multisignate structure and the similarity between the single stranded forms (Fig. 3a and b) and double stranded forms (Fig. 3c and d) indicates that several chromophores are involved in inter-molecular and/or in intra-strand interactions. We have previously shown by SAXS^31^ and EPR spectroscopy^70^ that porphyrin-DNA tends to associate in solution, and it was estimated that a minimum of six porphyrins interact through a combination of intra- and intermolecular interactions, having an average centre-to-centre distance of 6.5–8.9 Å.^70^ Here, this is also demonstrated by recording the CD spectra of selected systems in various salt concentrations (0 M, 0.1 M and 1.0 M NaCl), where a low salt concentration should suppress intermolecular interactions (Fig. 3e). The spectra clearly change upon increasing salt concentration; however, even in pure water the intermolecular interactions persist owing to the high hydrophobicity of the porphyrins. For example the multiple CD bands in the Soret region of **U1**·**F3**, which are more clearly seen in the presence of NaCl, point out that inter-duplex interactions are a result of the stacking and is more pronounced for the flexible linker. Similar multiple CD bands due to porphyrin–porphyrin stacking in inter-duplex aggregates have been described by Berova *et al.* ^53^ The phenomenon of intermolecular interactions can also be seen with pyrenes,^71,72^ perylenes^73,74^ or other porphyrin constructs.^35,75^

Particularly interesting are the strands **R4** and **F4** containing two porphyrins, which do show differences going from ssODN to dsODN forms, since a combination of intra- and intermolecular interactions determines the shape of the overall CD profile. The differences between the single and double stranded forms demonstrate the sensitivity of CD spectroscopy, which allows to distinguish between the intramolecular 1,3-porphyrin interactions and the intermolecular stacking. Comparing the **R** with the **F** series confirms that the added flexibility of the linker induces different orientations of the porphyrins, giving a better overlap of the π-systems which is seen in the larger bisignate bands of the spectra.

Similar trends can be seen in the zipper arrays **R**·**R** and **F**·**F**. Both the **R**- and the **F**-series show similar CD spectra (Fig. 3c and d), indicating that the porphyrins adopt a similar chiral twist. In the **F**-series, the porphyrins clearly act as circular oscillators since due to the restricted rotation around the bond between the porphyrin and the functionalized phenyl group both Soret components B_*x*_ and B_*y*_ are given equal weight.^76^ This results into complex CD bands consisting of multiple exciton couplet contributions; the porphyrins seem to be closer aligned due to the added flexibility of the linker. Exceptions are the systems **R1**·**R4** and **F1**·**F4**, where the CD signals do not appear as a superposition of the +1 or –1 interstrand zipper-arrangements. We speculate that the sterical demand of the porphyrins distorts the array formation. Mixed arrays **R**·**F** and **F**·**R** have more distorted CD signatures as can be seen from Fig. 3f. The arrays **R1**·**F2** and **R1**·**F3** show similar signatures in the spectra and their porphyrin arrangement seems comparable; the same holds for the arrays **R1**·**F4** and **F1**·**R4**. These arrays also have Δ*T*_m_ values in a similar range (Table 1). Whilst it is not possible to deduce the structure of the arrangement from the CD spectra, the overall picture does confirm that the type of linker (acetylene *vs.* propargyl-amide) as well as the sequence context strongly influences the intramolecular interactions and leads to a different orientation of the porphyrins in the major groove of the DNA.^31^ Particularly the use of mixed linker systems seems advantageous in terms of forming more evenly distributed porphyrin stacks.

### Circular dichroism of zinc metallated LNA strands

**Fig. 4:**
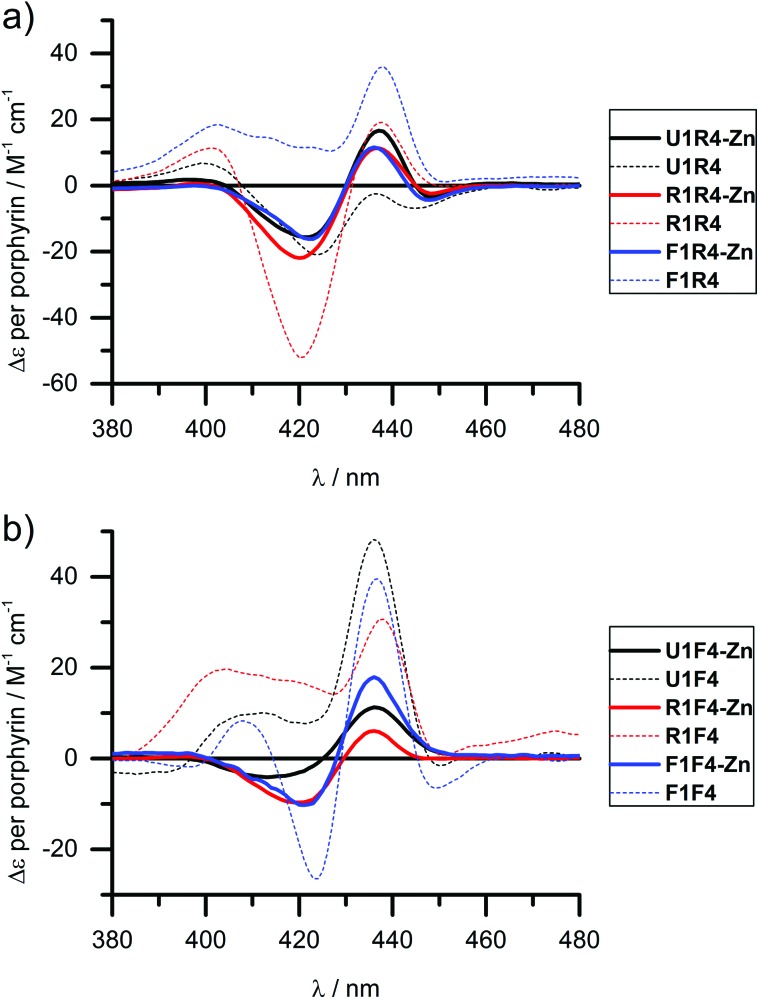
CD spectra of zinc metallated porphyrin arrays, containing either (a) **R4**-Zn or (b) **F4**-Zn, in comparison with the non-metallated duplexes. Δ*ε* values are given per porphyrin (normalized to the number of porphyrins present). Conditions as in Fig. 2.

We have shown previously that the porphyrins can be metallated easily with zinc, cobalt or copper when attached to DNA.^31,40^ This works equally well in the case of LNA, though we have only explored zinc in this respect and made **R4-Zn** and **F4-Zn**, respectively. The CD spectra of arrays containing the Zn-LNA and either **U1**, **R1** or **F1** as a complementary strand show that the exciton coupling signatures are quite similar in duplexes containing either **R4-Zn** or **F4-Zn** (Fig. 4) and are distinctively different to their non-metallated counterparts (apart **U1**·**R4** and **U1**·**R4-Zn**). Zn porphyrins that are attached *via* rigid linker clearly interact more prominently with each other in **R4-Zn**. They therefore exhibit a characteristic CD spectrum as in **U1**·**R4-Zn**. In comparison with **U1**·**R4**, the Zn analogue possesses not only added planarity and symmetry, but is also more hydrophilic due to axially coordinated water, and should therefore be less prone to aggregation in the presence of NaCl. In **R1**·**R4-Zn** three rigid porphyrins are available for interaction, but the CD profile seems to exclude the middle rigid porphyrin from interaction, as indicated by the similarity with **U1**·**R4-Zn**, but this is difficult to predict without a precise 3D model. The same is observed for **F1**·**R4-Zn**. Therefore, all the three CD profiles appear very similar, showing pair-wise porphyrin–porphyrin interactions which is independent on the array composition, and the systems give very similar Δ*ε* values per porphyrin for all duplexes. Clearly, the increased flexibility of the **F**-type porphyrin is beneficial for exciton coupling interactions, though the CD spectra look similar to the ones obtained for the **R**-series. However, the **F**-series is far more sensitive to changes in the system with changing Δ*ε* values per porphyrin for the three different duplexes. Here the differences to the non-metallated counterparts are more striking and indicate very different interactions.

## Conclusions

Porphyrins are building blocks for DNA based bio-nanotechnology with a wide range of applications, such as electron or energy transfer moieties and lipid anchors. The stability of DNA arrays containing multiple porphyrins is strongly influenced by the probe architecture, in particular single-strand modification *vs.* zipper arrangement. Here we have evaluated a porphyrin substituted LNA building block which inherently should give a more stable DNA duplex upon hybridization. We have shown that more stable arrays are formed when using three porphyrin-functionalized LNA monomers, compared to arrays based on the natural 2′-deoxyuridine derived building blocks, which is very promising for the formation of stable higher-ordered systems. Both thermodynamic data and CD spectra are complex and show a large dependency on the nature of the linker to the porphyrin and on the architecture of the porphyrin array. General trends indicate that: (i) modifying one DNA strands leads to either less ordered (acetylene linker) or more ordered (propargyl-amide linker) duplexes; (ii) the use of a rigid acetylene linker in a homo-porphyrin zipper-array leads to more ordered structures; (iii) the flexible linker induces less ordered structures in both homo- and hetero-porphyrinic zipper arrays, (iv) mixed zipper arrays are most stable and exceed the stability of the analogous dU-systems; (v) excitonic coupling between the porphyrins is strongly influenced by the nature of the linker and the sequence context but correlates well with the thermodynamic analysis; (vi) the flexible (and longer) linker allows for a better inter-strand interaction and formation of porphyrin-DNA clusters leading to more complex CD signatures. The detailed structure of the DNA and of the porphyrin arrangements in the modified region are not accessible yet, but clearly is affected by the parameters mentioned; the data therefore are valuable to gain insight into relative stability and electronic interactions in such systems.

## Author contributions

The manuscript was written through contributions of all authors.

## References

[cit1] Stulz E. (2012). Chem. – Eur. J..

[cit2] Song C., Wang Z. G., Ding B. Q. (2013). Small.

[cit3] Chhabra R., Sharma J., Liu Y., Rinker S., Yan H. (2010). Adv. Drug Delivery Rev..

[cit4] Lin C., Liu Y., Yan H. (2009). Biochemistry.

[cit5] Seeman N. C. (2003). Nature.

[cit6] Bath J., Turberfield A. J. (2007). Nat. Nanotechnol..

[cit7] Stulz E., Clever G., Shionoya M., Mao C. (2011). Chem. Soc. Rev..

[cit8] Schreiber R., Do J., Roller E. M., Zhang T., Schuller V. J., Nickels P. C., Feldmann J., Liedl T. (2014). Nat. Nanotechnol..

[cit9] Griffith A., Bandy T. J., Light M., Stulz E. (2013). Chem. Commun..

[cit10] Burns J. R., Zekonyte J., Siligardi G., Hussain R., Stulz E. (2011). Molecules.

[cit11] Malinovskii V. L., Wenger D., Häner R. (2010). Chem. Soc. Rev..

[cit12] Ensslen P., Wagenknecht H. A. (2015). Dalton Trans..

[cit13] Lindegaard D., Madsen A. S., Astakhova I. V., Malakhov A. D., Babu B. R., Korshun V. A., Wengel J. (2008). Bioorg. Med. Chem..

[cit14] Kool E. T. (2002). Acc. Chem. Res..

[cit15] Chelliserrykattil J., Lu H., Lee A. H. F., Kool E. T. (2008). ChemBioChem.

[cit16] Malyshev D. A., Dhami K., Lavergne T., Chen T. J., Dai N., Foster J. M., Correa I. R., Romesberg F. E. (2014). Nature.

[cit17] Menacher F., Wagenknecht H. A. (2011). Eur. J. Org. Chem..

[cit18] Vybornyi M., Nussbaumer A. L., Langenegger S. M., Häner R. (2014). Bioconjugate Chem..

[cit19] Bandy T. J., Brewer A., Burns J. R., Marth G., Nguyen T., Stulz E. (2011). Chem. Soc. Rev..

[cit20] Rubner M. M., Holzhauser C., Bohlander P. R., Wagenknecht H. A. (2012). Chem. – Eur. J..

[cit21] Probst M., Langenegger S. M., Häner R. (2014). Chem. Commun..

[cit22] Roethlisberger P., Wojciechowski F., Leumann C. J. (2013). Chem. – Eur. J..

[cit23] Teo Y. N., Kool E. T. (2012). Chem. Rev..

[cit24] Dziuba D., Pohl R., Hocek M. (2015). Chem. Commun..

[cit25] Palecek E., Tkac J., Bartosik M., Bertok T., Ostatna V., Palecek J. (2015). Chem. Rev..

[cit26] Bouamaied I., Fendt L. A., Wiesner M., Häussinger D., Amiot N., Thöni S., Stulz E. (2006). Pure Appl. Chem..

[cit27] Bouamaied I., Stulz E. (2005). Chimia.

[cit28] Bouamaied I., Stulz E. (2004). Synlett.

[cit29] Bouamaied I., Fendt L. A., Häussinger D., Wiesner M., Thöni S., Amiot N., Stulz E. (2007). Nucleosides, Nucleotides Nucleic Acids.

[cit30] Fendt L. A., Bouamaied I., Thöni S., Amiot N., Stulz E. (2007). J. Am. Chem. Soc..

[cit31] Brewer A., Siligardi G., Neylon C., Stulz E. (2011). Org. Biomol. Chem..

[cit32] Bouamaied I., Nguyen T., Rühl T., Stulz E. (2008). Org. Biomol. Chem..

[cit33] Nguyen T., Brewer A., Stulz E. (2009). Angew. Chem., Int. Ed..

[cit34] Endo M., Fujitsuka M., Majima T. (2008). Tetrahedron.

[cit35] Stephenson A. W. I., Partridge A. C., Filichev V. V. (2011). Chem. – Eur. J..

[cit36] Morales-Rojas H., Kool E. T. (2002). Org. Lett..

[cit37] Sitaula S., Reed S. M. (2008). Bioorg. Med. Chem. Lett..

[cit38] Balaz M., Holmes A. E., Benedetti M., Proni G., Berova N. (2005). Bioorg. Med. Chem..

[cit39] Balaz M., Bitsch-Jensen K., Mammana A., Ellestad G. A., Nakanishi K., Berova N. (2007). Pure Appl. Chem..

[cit40] Mammana A., Asakawa T., Bitsch-Jensen K., Wolfe A., Chaturantabut S., Otani Y., Li X. X., Li Z. M., Nakanishi K., Balaz M., Ellestad G. A., Berova N. (2008). Bioorg. Med. Chem..

[cit41] Balaz M., Holmes A. E., Benedetti M., Rodriguez P. C., Berova N., Nakanishi K., Proni G. (2005). J. Am. Chem. Soc..

[cit42] Stephenson A. W. I., Bomholt N., Partridge A. C., Filichev V. V. (2010). ChemBioChem.

[cit43] Wellner C., Wagenknecht H. A. (2014). Org. Lett..

[cit44] Balaz M., Li B. C., Jockusch S., Ellestad G. A., Berova N. (2006). Angew. Chem., Int. Ed..

[cit45] Balaz M., Steinkruger J. D., Ellestad G. A., Berova N. (2005). Org. Lett..

[cit46] Balaz M., Li B. C., Steinkruger J. D., Ellestad G. A., Nakanishi K., Berova N. (2006). Org. Biomol. Chem..

[cit47] Endo M., Fujitsuka M., Majima T. (2008). J. Org. Chem..

[cit48] Onoda A., Igarashi M., Naganawa S., Sasaki K., Ariyasu S., Yamamura T. (2009). Bull. Chem. Soc. Jpn..

[cit49] Berlin K., Jain R. K., Simon M. D., Richert C. (1998). J. Org. Chem..

[cit50] Le Doan T., Praseuth D., Perrouault L., Chassignol M., Thuong N. T., Helene C. (1990). Bioconjugate Chem..

[cit51] Murashima T., Hayata K., Saiki Y., Matsui J., Miyoshi D., Yamada T., Miyazawa T., Sugimoto N. (2007). Tetrahedron Lett..

[cit52] Li H. D., Fedorova O. S., Trumble W. R., Fletcher T. R., Czuchajowski L. (1997). Bioconjugate Chem..

[cit53] Mammana A., Pescitelli G., Asakawa T., Jockusch S., Petrovic A. G., Monaco R. R., Purrello R., Turro N. J., Nakanishi K., Ellestad G. A., Balaz M., Berova N. (2009). Chem. – Eur. J..

[cit54] Berova N., Bari L. D., Pescitelli G. (2007). Chem. Soc. Rev..

[cit55] Balaz M., De Napoli M., Holmes A. E., Mammana A., Nakanishi K., Berova N., Purrello R. (2005). Angew. Chem., Int. Ed..

[cit56] Grabowska I., Singleton D. G., Stachyra A., Gora-Sochacka A., Sirko A., Zagorski-Ostoja W., Radecka H., Stulz E., Radecki J. (2014). Chem. Commun..

[cit57] Endo M., Seeman N. C., Majima T. (2005). Angew. Chem., Int. Ed..

[cit58] Burns J. R., Stulz E., Howorka S. (2013). Nano Lett..

[cit59] Burns J. R., Gopfrich K., Wood J. W., Thacker V. V., Stulz E., Keyser U. F., Howorka S. (2013). Angew. Chem., Int. Ed..

[cit60] Woller J. G., Hannestad J. K., Albinsson B. (2013). J. Am. Chem. Soc..

[cit61] Kaur H., Babu B. R., Maiti S. (2007). Chem. Rev..

[cit62] Varghese R., Wagenknecht H. A. (2009). Chem. Commun..

[cit63] Guenther D. C., Kumar P., Anderson B. A., Hrdlicka P. J. (2014). Chem. Commun..

[cit64] Østergaard M. E., Kumar P., Baral B., Guenther D. C., Anderson B. A., Ytreberg F. M., Deobald L., Paszczynski A. J., Sharma P. K., Hrdlicka P. J. (2011). Chem. – Eur. J..

[cit65] Kumar P., Østergaard M. E., Baral B., Anderson B. A., Guenther D. C., Kaura M., Raible D. J., Sharma P. K., Hrdlicka P. J. (2014). J. Org. Chem..

[cit66] McTigue P. M., Peterson R. J., Kahn J. D. (2004). Biochemistry.

[cit67] Hussain R., Javorfi T., Siligardi G. (2012). J. Synchrotron Rad..

[cit68] Datta K., Johnson N. P., von Hippel P. H. (2010). Proc. Natl. Acad. Sci. U. S. A..

[cit69] Romainczyk O., Endeward B., Prisner T. F., Engels J. W. (2011). Mol. BioSyst..

[cit70] Nguyen T., Hakansson P., Edge R., Collison D., Goodman B. A., Burns J. R., Stulz E. (2014). New J. Chem..

[cit71] Doluca O., Withers J. M., Loo T. S., Edwards P. J. B., Gonzalez C., Filichev V. V. (2015). Org. Biomol. Chem..

[cit72] Lee I. J., Kim B. H. (2012). Chem. Commun..

[cit73] Wagner C., Wagenknecht H. A. (2006). Org. Lett..

[cit74] Baumstark D., Wagenknecht H. A. (2008). Angew. Chem., Int. Ed..

[cit75] Sargsyan G., Balaz M. (2012). Org. Biomol. Chem..

[cit76] Pescitelli G., Gabriel S., Wang Y. K., Fleischhauer J., Woody R. W., Berova N. (2003). J. Am. Chem. Soc..

