# Supplementary material for: Increased duplex stabilization in porphyrin-LNA zipper arrays with structure dependent exciton coupling
Source: Org Biomol Chem. 2015 Sep 29;14(1):149–57. doi: 10.1039/c5ob01681a (PMC4766578; doi:10.1039/c5ob01681a)
Supplement: Supplementary file 1 [file OB-014-C5OB01681A-s001.pdf]

Electronic Supplementary Material (ESI) for Organic & Biomolecular Chemistry.
This journal is © The Royal Society of Chemistry 2015

Electronic Supporting Information

Increased duplex stabilization in porphyrin-LNA zipper arrays with structure dependent exciton coupling

Daniel G. Singleton[†], Rohanah Hussain[‡], Giuliano Siligardi,[‡] Pawan Kumar[§], Patrick J. Hrdlicka[§], Nina Berova[∫], and Eugen Stulz*[†]

† School of Chemistry and Institute for Life Sciences, University of Southampton, Highfield, Southampton, SO17 1BJ, UK. ‡ Diamond Light Source, Harwell Science and Innovation Campus, Didcot, Oxfordshire OX11 0DE, UK. § Department of Chemistry, University of Idaho, Moscow, ID 83844, United States. ∫ Department of Chemistry, Columbia University, 3000 Broadway, New York, NY 10027, United States.

General

Chemicals were supplied by Sigma-Aldrich, Link Technologies, Glen Research, and Berry and Associates and used as received. DNA purification columns were supplied by Glen Research and Berry and Associates. Column chromatography was carried out using silica gel (Kieselgel 60), silica gel type H and/or basic alumina (50-200 μm, Brockmann activity I). TLC was carried out on Merck aluminium backed sheets of silica gel 60 F_{254} or aluminium backed sheets of alumina 60 F_{254} and were visualised using UV light (254 nm and 365 nm), anisaldehyde, phosphomolybdic acid (5 % in ethanol), and potassium permanganate in water.

Proton and carbon NMR spectra were recorded at 300/400 MHz and 75/100 MHz, respectively, using either a Bruker AC300 or Bruker DPX400 spectrometer. Chemical shifts are given in ppm and spectra are calibrated to the residual solvent peak. Coupling constants (*J*) are given in Hertz (Hz). Assignment was aided by DEPT-135, ^{1}H–^{1}H COSY, HMQC and HMBC experiments and Lorentz-Gauss resolution enhancement data reprocessing (ACD/SpecManager 12.0, ACD Labs).

Low resolution electrospray mass spectrometry was conducted on a Waters ZMD. MALDI-TOF was conducted on a ThermoBioAnalysis Dynamo using a *p*-nitroaniline matrix and referenced against TPP (Mw – 614.25) and 2,8,12,18-tetrahexyl-3,7,13,17-tetramethyl-5,15-di(*p*-(3-hydroxy-3-methyl-but-2-ynyl)phenyl porphyrin (Mw – 1082.88) or using a Micromass TOFSpec2E using external calibrants of terfenadine, bradykinin, angiotensin 1, renin substrate and ACTH clip for masses under 5000 Da, whereas oligonucleotides ranging from 5000 to 15000 Da were used for larger molecules.

UV-vis spectroscopy experiments were conducted using a Varian Cary 300 Bio spectrophotometer, and fluorescence spectroscopy experiments were conducted using a Varian Cary Eclipse fluorescence spectrophotometer, using quartz cells (supplied by Hellma and Starna) with 1 mm, 2 mm or 1 cm path lengths. The temperature of experiments was controlled using a Varian Cary Temperature Controller and peltier system with a Varian Cary Series II Temperature Probe. Concentrations of oligonucleotides (ODNs) were calculated using the Beer-Lambert law for the absorption at 260 nm. Molar extinction coefficients were obtained by the molar extinction calculator provided by IDT and by replacing the appropriate thymidines with the value of the porphyrin-dU (ε_{260} = 13'460 L·mol^{-1}·cm^{-1}) as determined previously.[1]

CD spectroscopy was recorded using a Chirascan Plus, or on Diamond Light Source beamline B23 Station B. The data was recorded in mdeg and corrected to delta epsilon using the formula $\Delta\varepsilon = \theta$ / (10 x conc. x path length x 3298); conc. is in mol/litre, path length is in cm.

All spectra were recorded at 4 μM concentration single stranded ODN in phosphate buffer (50 mM sodium phosphate, 100 mM sodium chloride, 1 mM di-sodium EDTA, pH 7). Duplexes were formed by heating the samples to 85 °C for two minutes, followed by slow cooling (1 °C / min) to 20 °C. Stock solutions of HPLC-pure DNA were stored at 4 °C (no precipitation was observed). The ODNs were used at a concentration of 2.5 μM for melting analysis.

Synthesis of building blocks

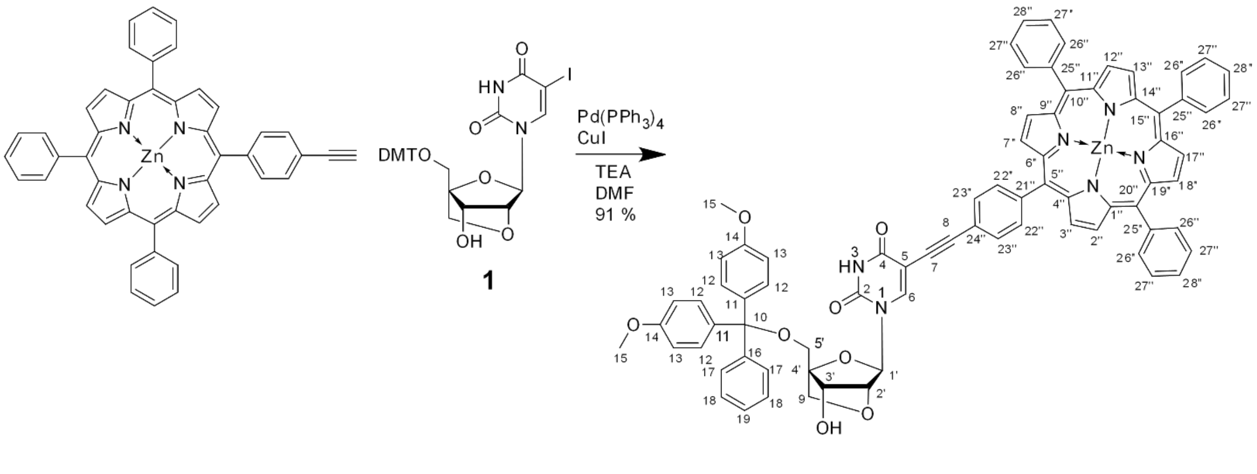

1

2

3

Using flame dried glassware under an inert atmosphere in the absence of light, zinc (II) 5–*p*-ethynylphenyl–10,15,20–triphenyl porphyrin **2**[2] (102 mg, 0.15 mmol, 1.02 eq.), 5’-*O*-(4,4’-dimethoxytrityl)-5-iodo-LNA-uridine **1**[3] (100 mg, 0.15 mmol, 1.00 eq.), copper(I) iodide (9 mg, 48 μmol, 0.33 eq.) and tetrakis(triphenylphosphine) palladium(0) (28 mg, 24 μmol, 0.17 eq.) were dissolved in DMF (3 mL). The reaction mixture was further purged with N_2 for 20 mins prior to the addition of triethylamine (40 μL, 0.44 mmol, 3.3 eq). The reaction was allowed to stir for 7 hr before being extracted into ethyl acetate (100 mL), and washed with 5 % w/v Na_2EDTA (pH = 9) (2 × 100 mL) and sat. NaCl (100 mL), dried (Na_2SO_4), and the solvent removed *in vacuo* after filtration and washing of the drying agent. The crude product was purified by column chromatography (silica containing 20 % silica H neutralised with TEA, eluent - DCM → DCM + 3 % MeOH). The pure product **3** was collected as a purple solid (167 mg, 0.13 mmol, 91 %).

$\mathbf{R}_f$ 0.9 (silica, DCM + 10 % MeOH)

^{1}H NMR (300 MHz, $CDCl_3$): δ = 9.03-9.10 (m, 6H, **H**$^{2''}$, **H**$^{8''}$, **H**$^{12''}$, **H**$^{13''}$, **H**$^{17''}$, **H**$^{18''}$),), 8.99-9.03 (m, 2H, **H**$^{3''}$, **H**$^{7''}$), 8.34 (m, 6H, **H**$^{26''}$), 8.19 (s, 1H, **H**^{6}), 8.13 (d, *J* = 7.7 Hz, 2H, **H**$^{23''}$), 7.76-7.89 (m, 9H, **H**$^{27''}$, **H**$^{28'''}$), 7.62 (d, *J* = 7.5 Hz, 2H, **H**$^{22''}$), 7.39-7.56 (m, 8H,**H**^{12}, **H**^{17}, **H**^{18}), 7.30 (t, *J* = 7.0 Hz, 1H, **H**^{19}), 6.80-6.90 (m, 4H, **H**^{13}), 5.24 (s, 1H, **H**$^{1'}$), 4.80 (m, 2H, **H**$^{2'}$, **H**$^{3'}$), 4.18 (s, 1H, **H**^{9}), 3.98 (s, 1H, **H**^{9}), 3.63 (s, 6H, **H**^{15}), 3.56 (d, *J* = 9.2 Hz, 2H, **H**$^{5'}$)

^{13}C NMR (100 MHz, $CDCl_3$): δ = 161.3 (CH, **C4**), 158.4 (CH, **C14**), 150.2 (C, **C5’’**, **C10’’**, **C15’’**, **C20’’**), 148.1 (C, **C2**), 144.2 (C, **C24’’**), 143.0 (C, **C16**), 142.8 (C, **C25’’**), 140.9 (CH, **C6**), 135.7 (C, **C11**), 134.5 (CH, **C26’’**), 134.3 (CH, **C23’’**), 132.0 (CH, **C2’’**, **C3’’**, **C7’’**, **C8’’**, **C12’’**, **C13’’**, **C17’’**, **C18’’**), 129.8 (CH, **C12**), 129.7 (CH, **C17**, CH, **C18**), 129.0 (CH, **C19**), 128.0 (CH, **C22’’**), 126.5 (CH, **C27’’**, **C28’’**), 113.4 (CH **C13**), 113.1 (CH **C13**), 100.0 (C, **C8**), 94.3 (C, **C7**), (CH, **C1’**), 86.6 (C, **C10**), 80.8 (C, **C5**), 78.6 (CH, **C2’**), 71.4 (CH_2, **C9**), 70.2 (CH, **C3’**), 58.5 (CH_2, **C5’**)

ESI negative ($C_{55}H_{38}N_6O_5Zn$): Calculated mass 1256.35, observed mass 1255.6 $[M-H]^-$

UV-Vis (DCM, 8 μM): λ_{max} (log ε) 420 nm (5.15), 548 nm (3.73), 588 nm (2.89)

Emission (DCM, 8 μM): λ_{ex} 420 nm, λ_{em} (rel int) 598 nm (1), 645 nm (0.91)

This report was created by ACD/NMR Processor Academic Edition. For more information go to www.acdlabs.com/nmrproc/

Rigid Porphyrin LNA monomer

15/09/2015 13:37:22

Formula $C_{77}H_{50}N_{6}O_{6}Zn$	*FW* 1258.7129

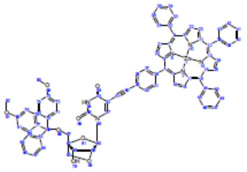

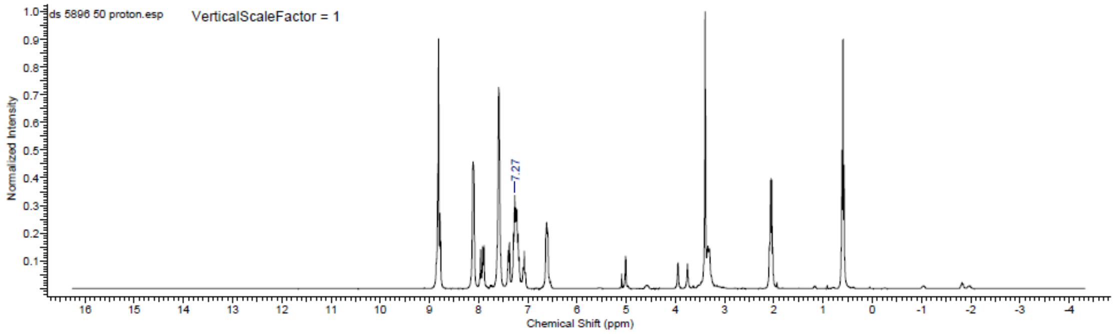

No.	(ppm)	Annotation	Layer No.	Created By	Created At	Modified By	Modified At
1	7.27	CHLOROFORM-d	1	ds504	Mon 01/11/2010 14:59:12		

No.	(ppm)	(Hz)	Height
1	7.27	2181.9	0.3376

This report was created by ACD/NMR Processor Academic Edition. For more information go to www.acdlabs.com/nmrproc/

Rigid Porphyrin LNA monomer

15/09/2015 13:38:21

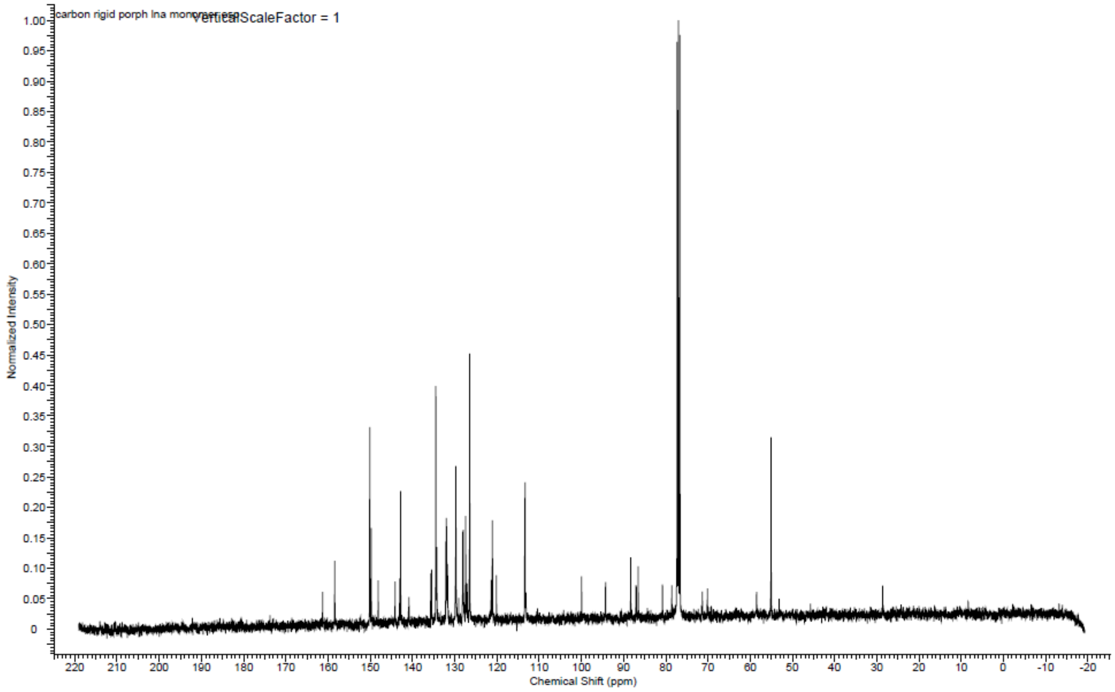

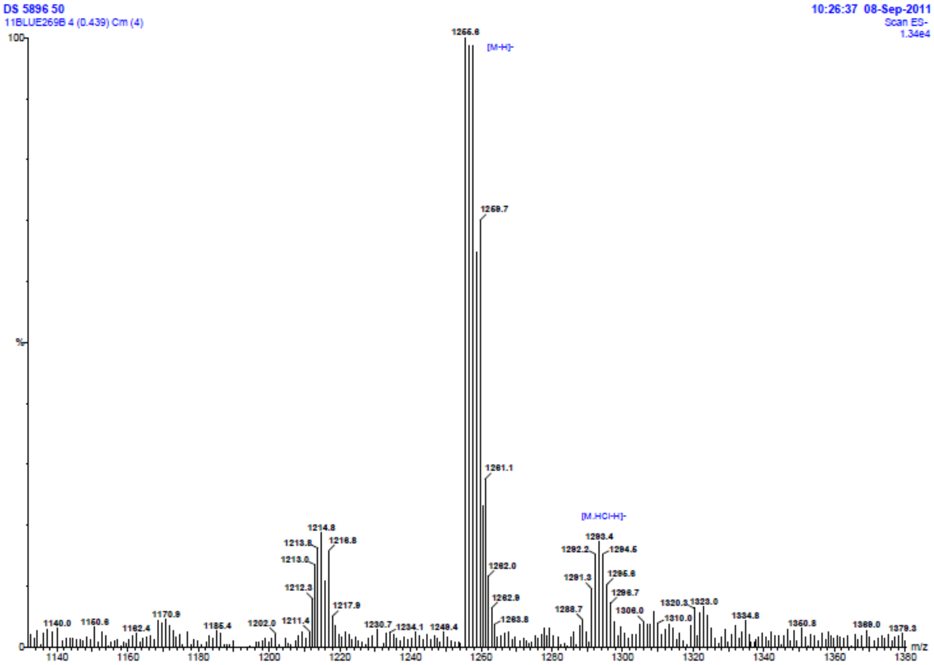

DS 5896 50
11BLUE269B 4 (0.439) Cm (4)
10:26:37 08-Sep-2011
Scan ES-
1.34e4
[M-H]-
[M.HCl-H]-
m/z

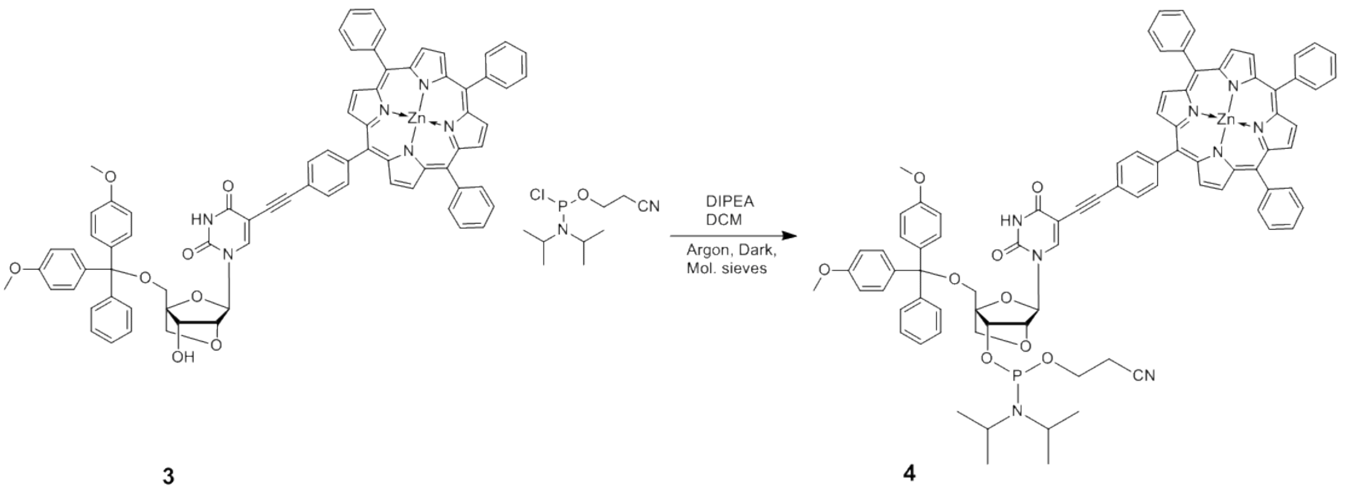

3 4

Using oven dried glassware under Schlenk conditions, 5’-DMT-5-(5’’*p*-ethynylphenyl-10’’,15’’,20’’-triphenyl-21’’,23’’-zinc (II) porphyrin)-U-LNA **3** (70 mg, 56 µmol, 1.0 eq.) was dissolved in anhydrous DCM (3 mL) with molecular sieves (3 Å) in the dark. Diisopropylethylamine (DIPEA, 39 µL, 0.22 mmol, 4.0 eq.) and 2-cyanoethyl N,N-diisopropylchlorophosphoramidite (CEP-Cl, 26 µL, 0.11 mmol, 2.0 eq.) were added and the reaction allowed to stir at room temperature for 2 hr. The reaction had not gone to completion in this time so additional DIPEA (39 µL, 0.22 mmol, 4.0 eq.) and CEP-C (26 µL, 0.11 mmol, 2.0 eq.) were added. The reaction was found to be complete after 3.5 hr. The crude product was precipitated form hexane (5 mL) and cooled (-18 °C) for 10 mins, hexane was decanted off and the crude product washed with further hexane (5 mL). The crude product **4** was used immediately for DNA synthesis.

$\mathbf{R}_f$ 0.7 (silica, DCM + 10 % MeOH + 0.5 % triethylamine)

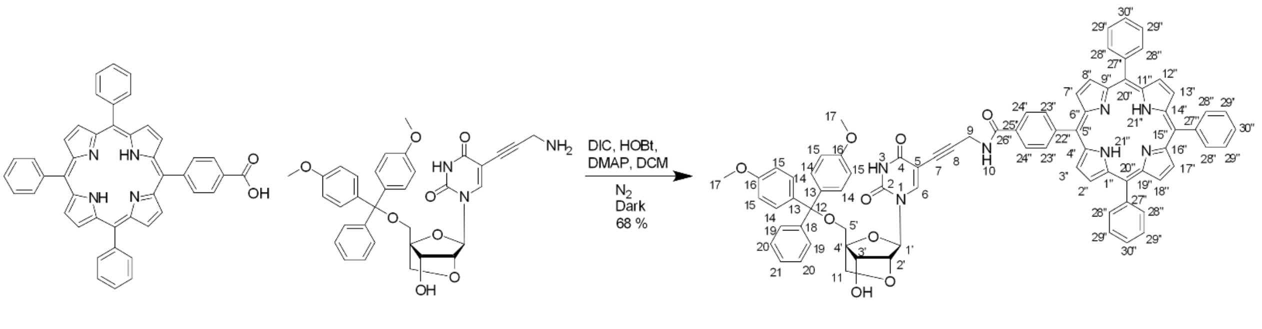

5 6 7

Porphyrin acid **5**[4] (113 mg, 0.17 mmol, 1.0 eq.) was dissolved in anhydrous DCM (5 mL). Propargyl uridine LNA **6**[5] (115 mg, 0.19 mmol, 1.1 eq.), diisopropyl-carbodiimide (53 µL, 0.34 mmol, 2.0 eq.), HOBt (26 mg, 0.30, 1.0 eq.) and DMAP (42 mg, 0.34 mmol, 2.0 eq.) were added to the purple reaction mixture and stirred in the dark under an inert atmosphere for 4 hr. The reaction mixture was diluted with DCM (25 mL) and washed with water (2 × 50 mL), sat. aq. KCl (50 mL), dried (Na_2SO_4) and the sol-

vent removed *in vacuo* after filtration and washing of the drying agent. The crude product was purified by column chromatography (silica neutralised with triethylamine, eluent – DCM → DCM + 2 % MeOH) to give the product **7** as a purple solid, (145 mg, 0.12 mmol, 68 %).

R_f 0.6 (silica, DCM + 10 % MeOH)

^{1}H NMR (400 MHz, $CDCl_3$): δ = 8.79-8.85 (m, 6H, **H$^{2''}$**, **H$^{8''}$**, **H$^{12''}$**, **H$^{13''}$**, **H$^{17''}$**, **H$^{18''}$**), 8.72 (m, 2H, **H$^{3''}$**, **H$^{4''}$**), 8.19 (d, *J* = 7.7 Hz, 2H, **H$^{24''}$**), 8.15 (m, 7H, **H^{6}**, **H$^{28''}$**), 7.96 (d, *J* = 7.7 Hz, 2H, **H$^{23''}$**), 7.62-7.82 (m, 9H, **H$^{29''}$**, **H$^{30''}$**), 7.49 (d, *J* = 7.7 Hz, 2H, **H^{19}**), 7.38 (dd, *J* = 8.7, 2.8 Hz, 4H, **H^{14}**), 7.25-7.31 (m, 2H, **H^{20}**), 7.16 (t, *J* = 7.1 Hz, 1H, **H^{21}**), 6.81 (dd, *J* = 8.7, 3.2 Hz, 4H, **H^{15}**), 5.63 (s, 1H, **H$^{1'}$**), 4.63 (s, 1H, **H$^{2'}$**), 4.29-4.49 (m, 3H, **H$^{3'}$**, **H^{9}**), 3.93 (d, *J* = 8.1 Hz, 1H, **H^{11}**), 3.83 (d, *J* = 8.2 Hz, 1H, **H^{11}**), 3.64-3.67 (m, 1H, **H$^{5'}$**), 3.63 (s, 3H, **H^{17}**), 3.61 (s, 3H, **H^{17}**), 3.56 (d, *J* = 8.2 Hz, 1H, **H$^{5'}$**), -2.78 (br. s., 2H, **H$^{21''}$**)

^{13}C NMR (100 MHz, $CDCl_3$): δ = 167.2 (C, **C26''**), 162.3 (C, **C4**), 158.6 (C, **C16**), 148.8 (C, **C2**), 145.6 (C, **C25''**), 144.5 (C, **C18**), 142.0 (CH, **C6**) 134.6 (C, **C13**), 134.5 (CH, **C24''**, **C28''**), 132.9 (C, **C27''**), 131.7 (CH, **C2''**, **C3''**, **C7''**, **C8''**, **C12''**, **C13''**, **C17''**, **C18''**), 130.1 (CH, **C14**), 128.0 (CH, **C20**), 127.7 (CH, **C19**), 126.7 (CH, **C29''**, **C30''**), 127.0 (CH, **C21**), 125.5 (CH, **C23''**), 113.5 (CH, **C15**), 87.5 (C, **C12**), 86.7 (CH, **C1'**), 79.23 (CH, **C2'**) 74.4 (CH_2 **C11**), 70.4 (CH, **C3'**), 58.6 (CH_2, **C5'**), 55.2 (CH_3, **C17**), 30.9 (CH_2, **C9**)

ESI Negative ($C_{79}H_{61}N_7O_9$): Calculated mass 1251.45, observed *m/z* 1250.7 $[M - H]^-$

UV-Vis (DCM, 2.4 µM): λ_{max} (log ε) 417 nm (5.68), 515 nm (4.33), 548 nm (3.99), 589 nm (3.83), 646 nm (3.64)

Emission (DCM, 2.4 µM): λ_{ex} 417 nm, λ_{em} (rel int) 650 nm (1), 717 nm (0.33)

This report was created by ACD/NMR Processor Academic Edition. For more information go to www.acdlabs.com/nmrproc/

Flexible Porphyrin LNA monomer

15/09/2015 13:31:20

Formula $C_{79}H_{61}N_{7}O_{9}$	*FW* 1252.3711

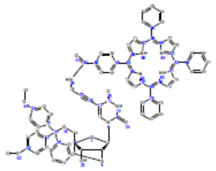

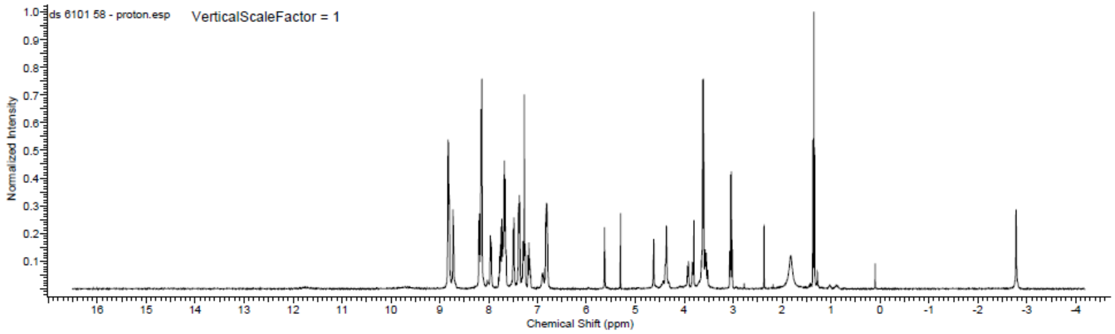

No.	(ppm)	Annotation	Layer No.	Created By	Created At	Modified By	Modified At
1	7.27	CHLOROFORM-d	1	ds504	Thu 08/09/2011 09:43:28		

This report was created by ACD/NMR Processor Academic Edition. For more information go to www.acdlabs.com/nmrproc/

Flexible Porphyrin LNA monomer

15/09/2015 13:40:26

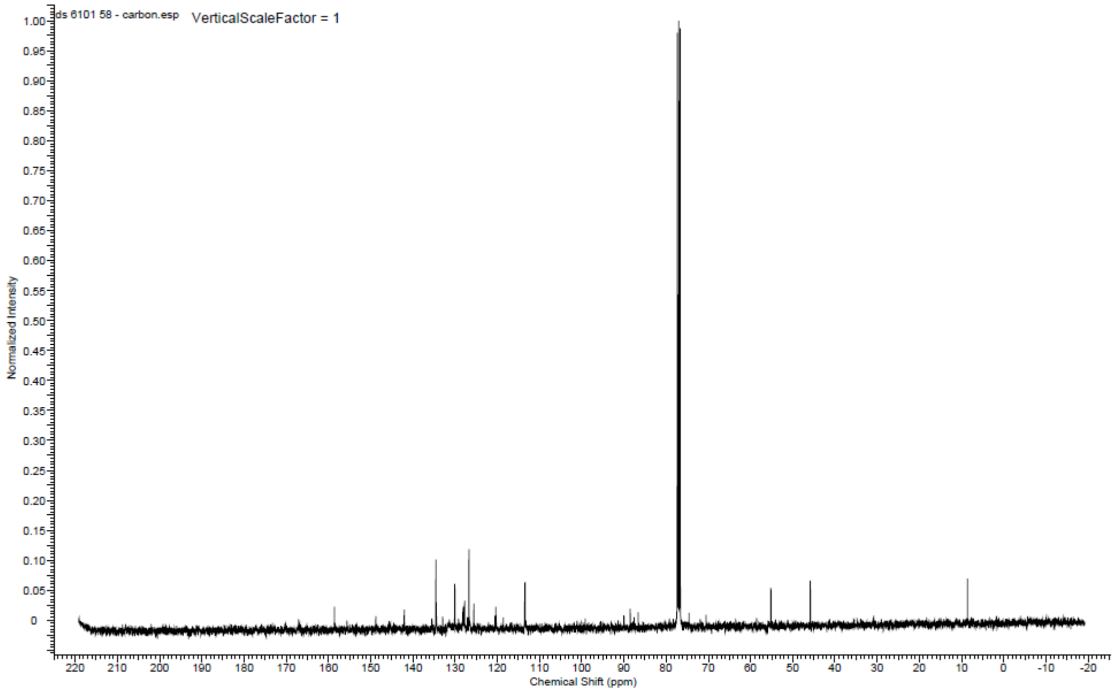

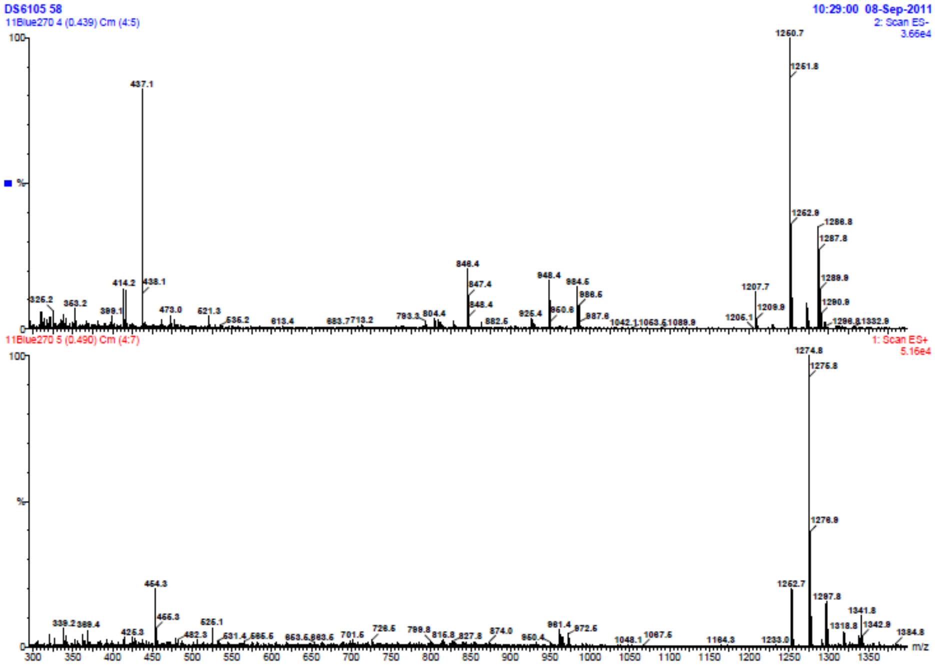
DS6105 58
11Blue270 4 (0.439) Cm (4:5)
10:29:00 08-Sep-2011
2: Scan ES-
3.66e4
437.1
1260.7
1261.8
1262.9
414.2
438.1
473.0
521.3
847.4
1207.7
1287.8
11Blue270 5 (0.490) Cm (4:7)
1: Scan ES+
5.16e4
1274.8
1275.8
454.3
455.3
525.1
1262.7
1341.8
1342.9
1384.8
1048.1
1067.5
1233.0
874.0
701.5
%
100
0
m/z
300
350
400
450
500
550
600
650
700
750
800
850
900
950
1000
1050
1100
1150
1200
1250
1300
1350

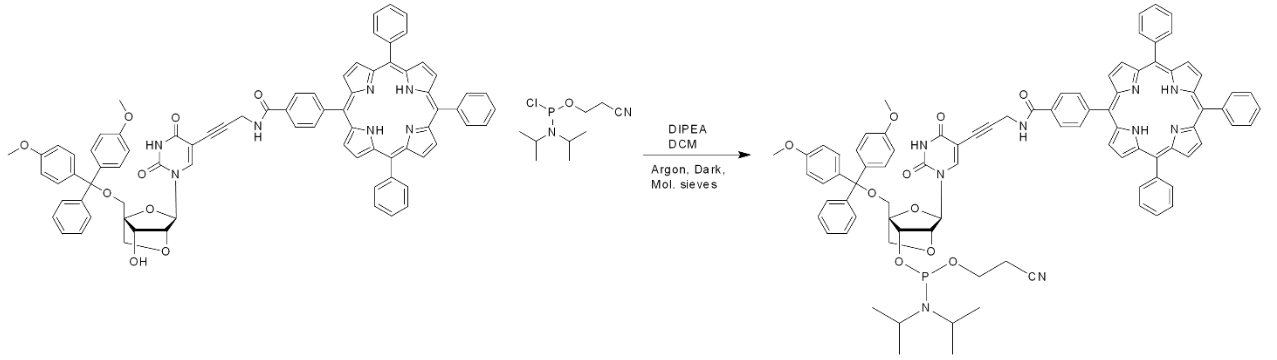

7 8

Using oven dried glassware under Schlenk conditions, *N*-(5'-DMT-5-propargyl-U-LNA)-5''-(*p*-benzamide)-10'',15'',20''-triphenyl-21''-*H*-23''-*H*-porphyrin **7** (103 mg, 82 µmol, 1.0 eq.) was dissolved in anhydrous DCM (3 mL) with molecular sieves (3 Å) in the dark. DIPEA (57 µL, 0.33 mmol, 4.0 eq.) and CEP-Cl (58 µL, 0.25 mmol, 3.0 eq.) were added and the reaction allowed to stir at room temperature for 1.75 hr. The crude product was precipitated form hexane (5 mL) and cooled (-18 °C) for 10 mins, hexane was decanted off and the crude product washed with further hexane (5 mL). The crude product **8** was used immediately for DNA synthesis.

R_f 0.7 (silica, DCM + 10 % MeOH + 0.5 % triethylamine)

DNA synthesis

DNA synthesis was carried out on an Applied Biosystems Expedite machine using 500 Å pore CPG beads. DNA synthesis reagents (SAFC or Link Technologies) were used as received. Deblocking steps used 3 % TCA in DCM solution, activation steps use 0.1 M 'Activator 42' (5-(*bis*-3,5-trifluoromethylphenyl)-1*H*-tetrazole) in MeCN, capping steps used acetic anhydride in THF (Cap A) and pyridine and NMI in THF (Cap B), oxidizing steps used 0.02 M iodine, pyridine and water in THF, washing steps used MeCN. Cleavage of the oligonucleotide from the solid support was achieved by passing concentrated ammonium hydroxide through the column using two syringes at either end for one hour at room temperature. This solution was then heated to 40 °C overnight to deprotect the oligonucleotide. Synthesis was conducted DMT-on.

Rigid alkyne linked porphyrin LNA phosphoramidite **4** (81 mg, 55.6 µmol) or flexible amide linked porphyrin LNA phosphoramidite **8** (119 mg, 82 µmol) was dissolved in DCM:MeCN (1:1, 1.5 mL, 37 mM or 3.0 mL, 27 mM) and coupled using an extended coupling time of 5 minutes in the DNA synthesiser; the DNA synthesis was conducted on a 1.0 µmol scale. The final base contained a fluorous tagged DMT (FDMT) for affinity purification.

FDMT-on oligonucleotide was diluted with an equal volume of loading buffer (Berry & Associates) and purified on Fluoro-Pak™ II according to the protocol from the manufacturer. Final analysis for purity was performed using RP-HPLC which was carried out using a Varian Galaxie system using a Waters XBridge OST C18, 2.5 µm 4.6 x 50mm column. Eluents used were 8.6 mM TEA / 100 mM HFIP buffer and MeOH. Eluents were filtered through a Supelco Nylon 66 Membrane filter (0.45 µm pore size) before use. Flow rates were set to 1 mL min^{-1}.

Strand	Sequence	Calculated ε_{260} ($mol^{-1}\ dm^{3}\ cm^{-1}$)	Yield (nmoles)
U1	5' – GTG ATA ACG – 3'	89900	611
U2	5' – GCA TAT CAC – 3'	88000	576
R1	5' – GTG A**4**A TGC – 3'	89900	562
R2	5' – GCA TA**4** CAC – 3'	88000	347
R3	5' – GCA **4**AT CAC – 3'	88000	644
R4	5' – GCA **4**A**4** CAC – 3'	88000	708
F1	5' – GTG A**8**A TGC – 3'	89900	228
F2	5' – GCA TA**8** CAC – 3'	88000	362
F3	5' – GCA **8**AT CAC – 3'	88000	371
F4	5' – GCA **8**A**8** CAC – 3'	88000	342

Melting analysis
UV-melting curves were obtained at 260 nm, 0.1 °C / min. The curves were fitted using Origin software; details can be found in the associated files available at http://dx.doi.org/10.5258/SOTON/381422.

The calculation takes the thermodynamic equilibria and the slopes at the beginning and end of the melting into account, using the following equation:

K_{eq}= (exp((-H/8.831*(x+273.15))+(S/8.831)))

where H = ΔH and S = ΔS, x = temperature in °C.

The absorbance y can be fitted as:

y = 0.5*((1-a)*(ss*(x-T_{max})+2*A_{max})+(a*(ds*(x-T_{min})+2*A_{min})))

where ss is the slope at high temp (ssDNA) and ds is the slope at low temp (dsDNA), A_{max}, A_{min}, T_{max} and T_{min} are maximal and minimal absorbances and temperatures, respectively. a is the fraction in duplex form given as:

a = (1+(1/(c*K_{eq}))*(1-sqrt(1+2*c*K_{eq})))

where c = concentration of the sample.

The equations were combined to give the fitting function as indicated in the examples below. c, R, T_{max} and T_{min} were held constant while all other parameters were allowed for variation until convergence of the fit.

UV melting curves

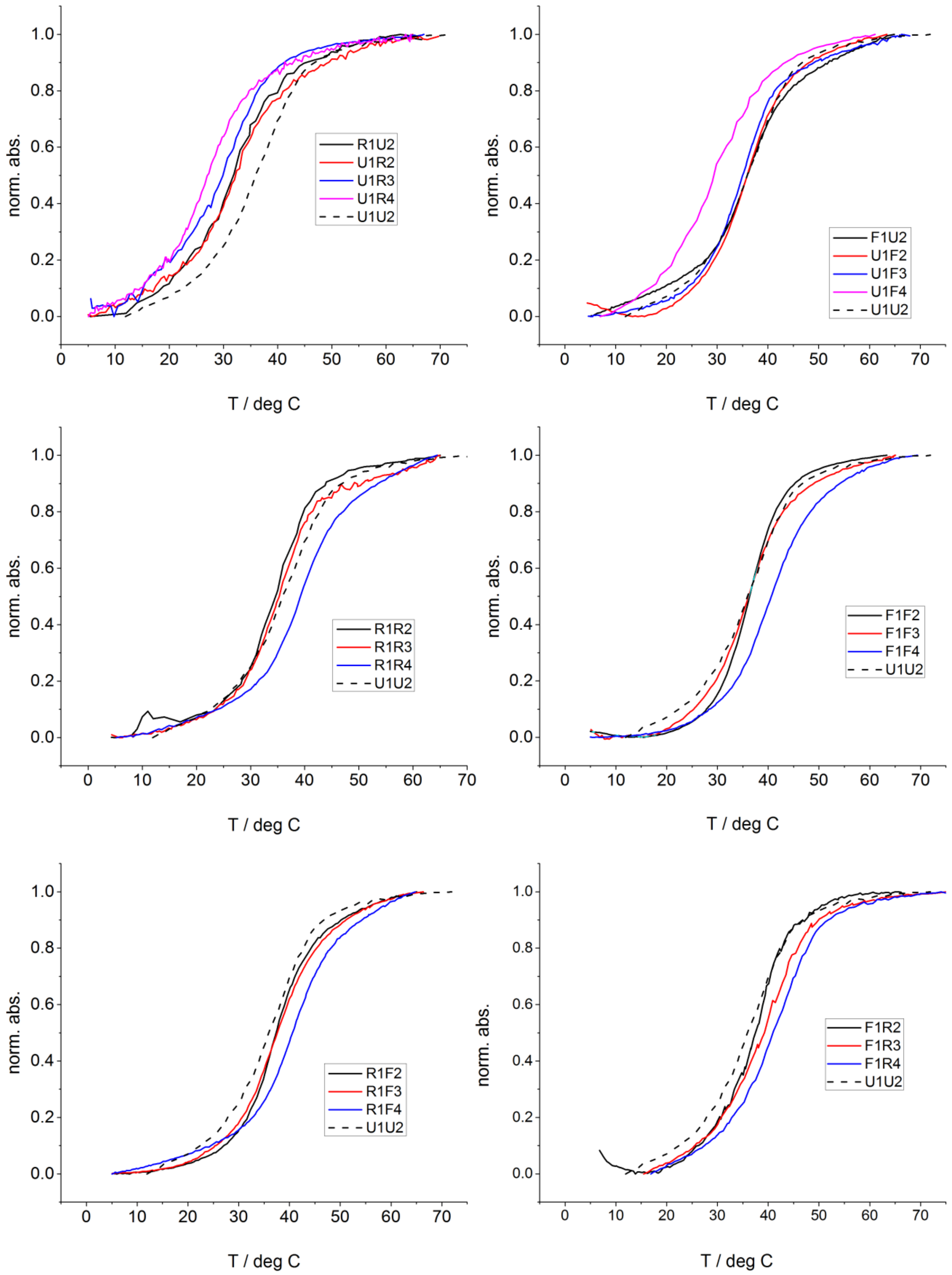

CD melting analysis

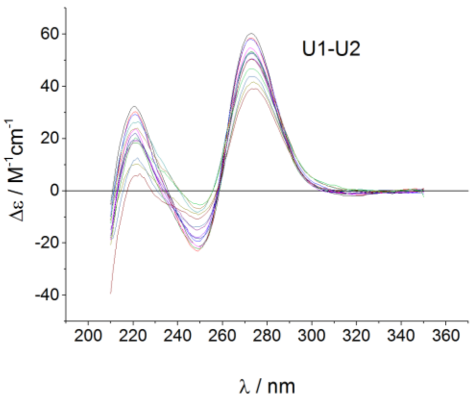

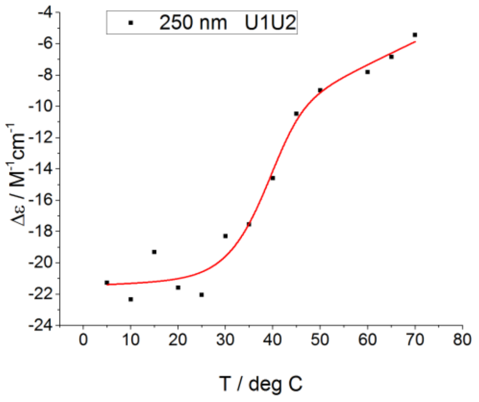

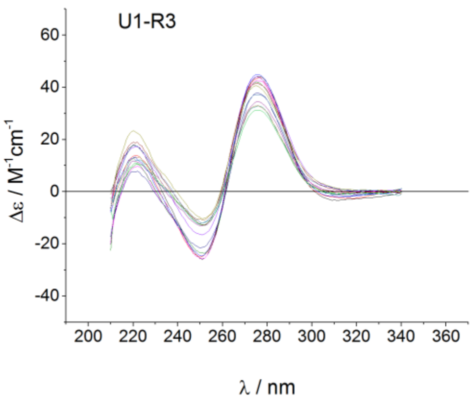

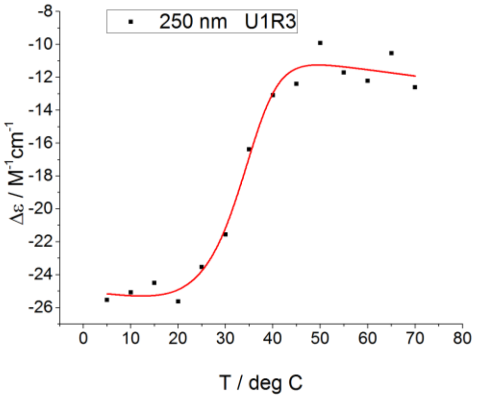

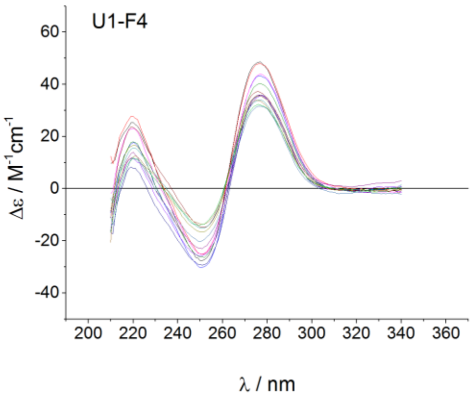

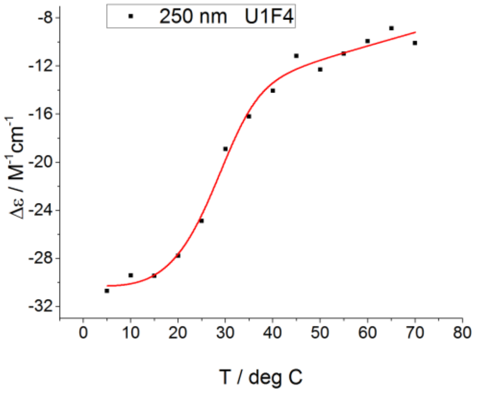

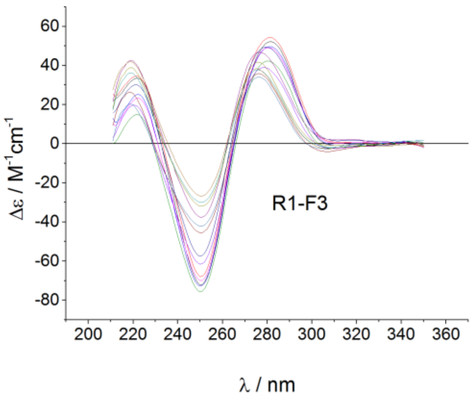

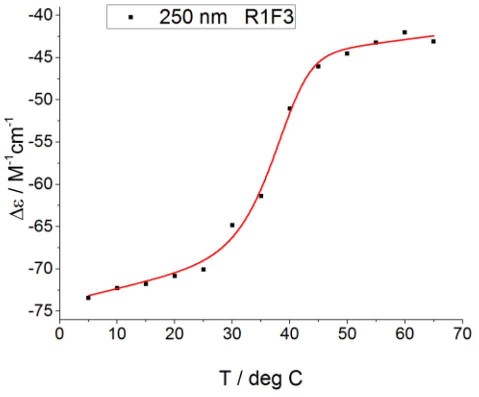

Examples of UV-melting curve fitting

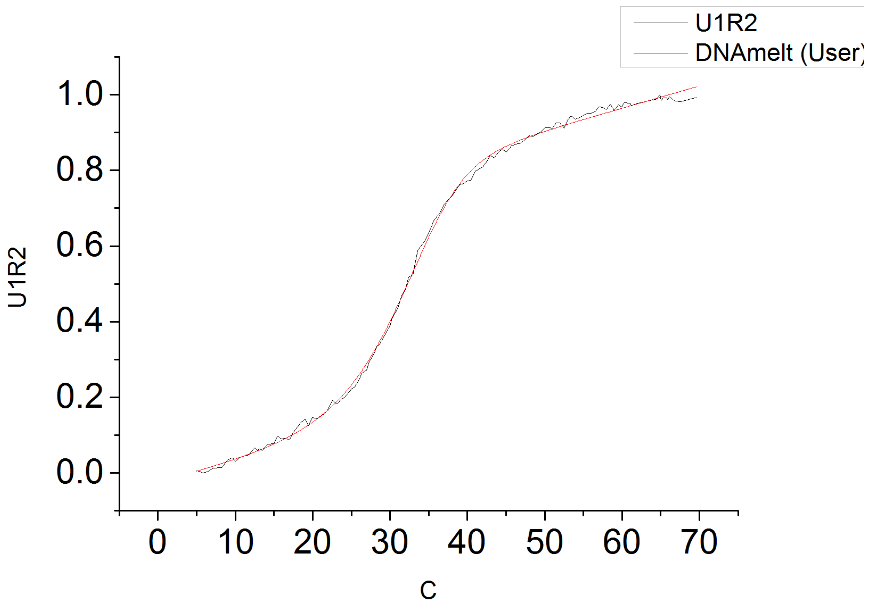

Model	DNAmelt (User)		
Equation	0.5*((1-(1+(1/(c*(EXP(-H/(R*(x+273.15))+S/R))))*(1-SQRT(1+2*c*(EXP(-H/(R*(x+273.15))+S/R))))))*(ss*(x-Tmax) +2*Amax)+((1+(1/(c*(EXP(-H/(R*(x+273.15))+S/R))))*(1-SQRT(1+2*c*(EXP(-H/(R*(x+273.15))+S/R)))))*(ds*(x-Tmin)+2*Amin)))		
Reduced Chi-Sqr	1.19059E-4		
Adj. R-Square	0.99915		
		Value	Standard Error
U1R2	H	-281044.99156	601.50205
U1R2	S	-802.97278	1.82522
U1R2	ss	0.01176	4.5472E-4
U1R2	ds	0.01125	9.8106E-4
U1R2	c	2.5E-6	0
U1R2	R	8.3155	0
U1R2	Tmax	70	0
U1R2	Tmin	5	0
U1R2	Amax	1.02305	0.00328
U1R2	Amin	0.00296	0.00389

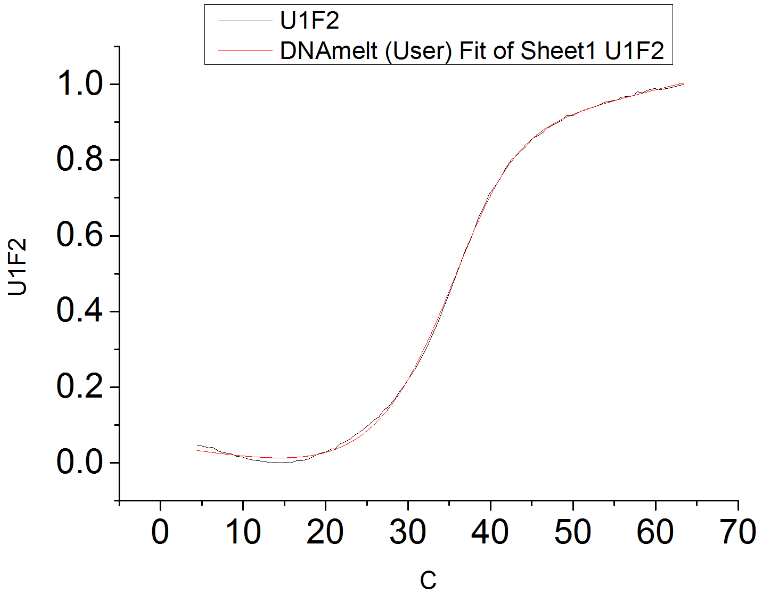

Model	DNAmelt (User)		
Equation	0.5*((1-(1+(1/(c*(EXP(-H/(R*(x+273.15))+S/R))))*(1-SQRT(1+2*c*(EXP(-H/(R*(x+273.15))+S/R))))))*(ss*(x-Tmax) +2*Amax)+((1+(1/(c*(EXP(-H/(R*(x+273.15))+S/R))))*(1-SQRT(1+2*c*(EXP(-H/(R*(x+273.15))+S/R)))))*(ds*(x-Tmin)+2*Amin)))		
Reduced Chi-Sqr	5.12702E-5		
Adj. R-Square	0.99968		
		Value	Standard Error
U1F2	H	-257149.99008	346.3784
U1F2	S	-719.13416	1.13629
U1F2	ss	0.01052	7.05249E-4
U1F2	ds	-0.00699	6.22667E-4
U1F2	c	2.5E-6	0
U1F2	R	8.3155	0
U1F2	Tmax	64	0
U1F2	Tmin	4.4	0
U1F2	Amax	1.00725	0.00325
U1F2	Amin	0.03096	0.00269

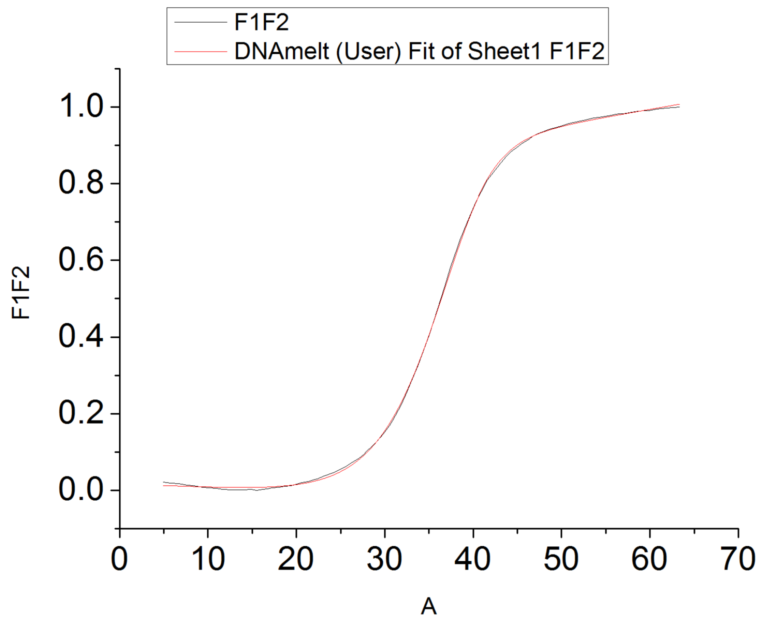

Model	DNAmelt (User)		
Equation	0.5*((1-(1+(1/(c*(EXP(-H/(R*(x+273.15))+S/R))))*(1-SQRT(1+2*c*(EXP(-H/(R*(x+273.15))+S/R))))))*(ss*(x-Tmax) +2*Amax)+((1+(1/(c*(EXP(-H/(R*(x+273.15))+S/R))))*(1-SQRT(1+2*c*(EXP(-H/(R*(x+273.15))+S/R)))))*(ds*(x-Tmin)+2*Amin)))		
Reduced Chi-Sqr	1.85627E-5		
Adj. R-Square	0.9999		
		Value	Standard Error
F1F2	H	-344277.40176	154.83404
F1F2	S	-996.68653	0.50644
F1F2	ss	0.00806	3.66998E-4
F1F2	ds	-0.00189	2.86541E-4
F1F2	c	2.5E-6	0
F1F2	R	8.3145	0
F1F2	Tmax	63	0
F1F2	Tmin	5	0
F1F2	Amax	1.00593	0.00175
F1F2	Amin	0.01246	0.0016

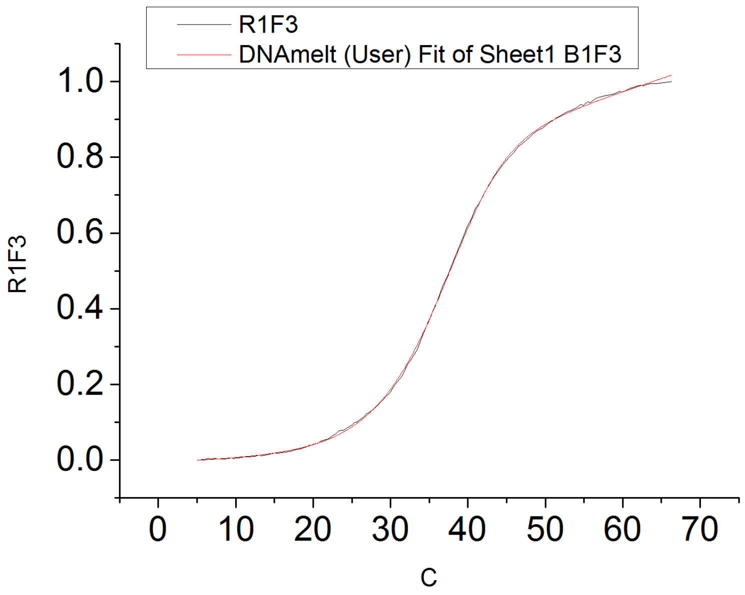

Model	DNAmelt (User)		
Equation	0.5*((1-(1+(1/(c*(EXP(-H/(R*(x+273.15))+S/R))))*(1-SQRT(1+2*c*(EXP(-H/(R*(x+273.15))+S/R))))))*(ss*(x-Tmax) +2*Amax)+((1+(1/(c*(EXP(-H/(R*(x+273.15))+S/R))))*(1-SQRT(1+2*c*(EXP(-H/(R*(x+273.15))+S/R)))))*(ds*(x-Tmin)+2*Amin)))		
Reduced Chi-Sqr	2.07029E-5		
Adj. R-Square	0.99987		
		Value	Standard Error
R1F3	H	-269660.52378	213.32388
R1F3	S	-753.27659	0.70142
R1F3	ss	0.01374	3.66218E-4
R1F3	ds	0.00212	2.98389E-4
R1F3	c	2.5E-6	0
R1F3	R	8.3155	0
R1F3	Tmax	66.4	0
R1F3	Tmin	5	0
R1F3	Amax	1.01777	0.0017
R1F3	Amin	-0.00144	0.00146

CD spectra
Single stranded porphyrin-LNA

all samples 20 °C, 4 µM, water, pH 7, 100 mM NaCl, 50 mM phosphate buffer

1) rigid linker

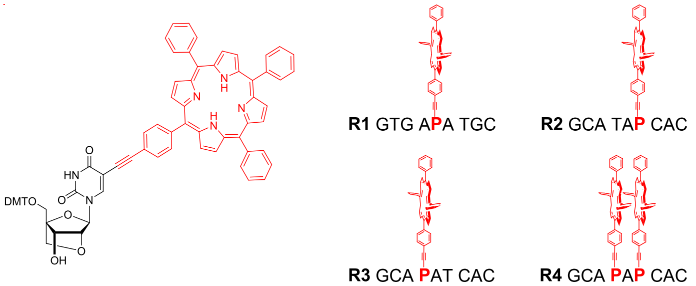

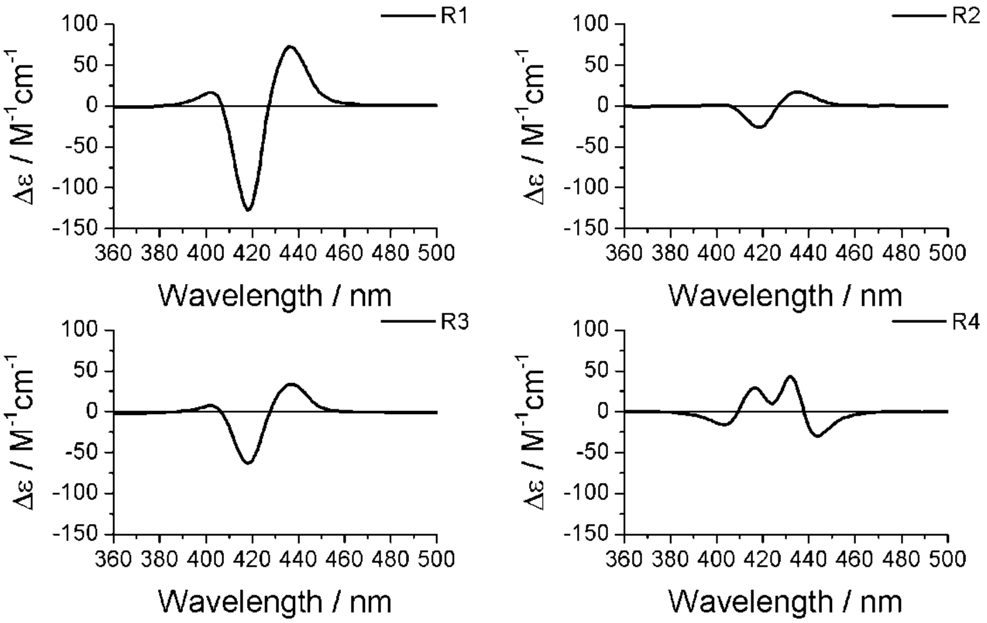

2) flexible linker

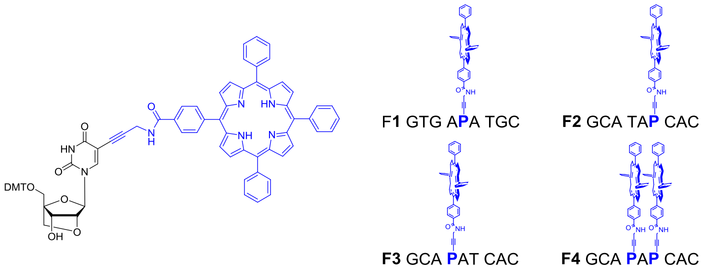

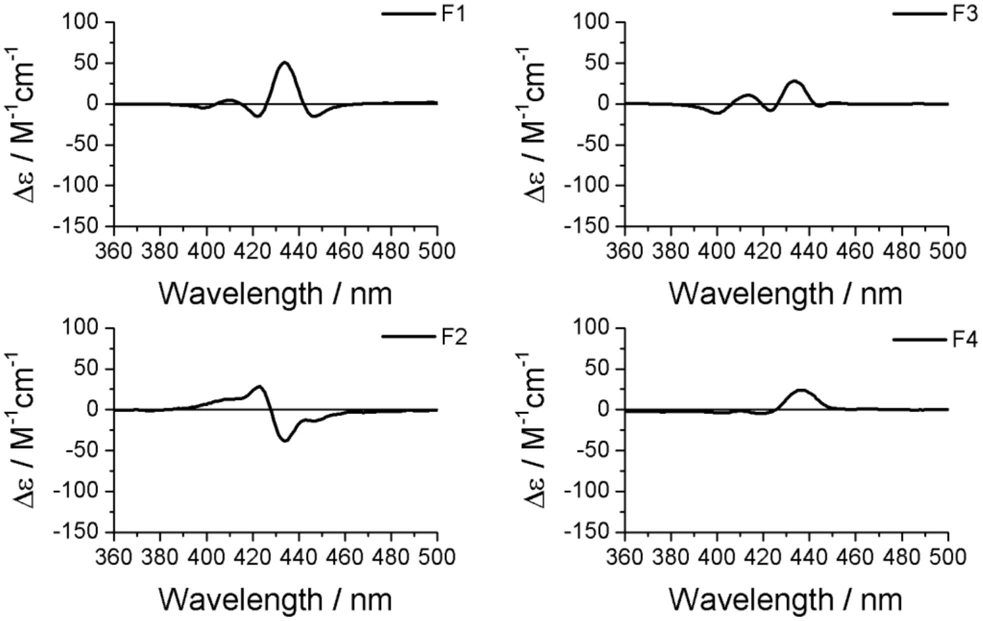

Double stranded porphyrin-LNA

3) rigid linker with unmodified complementary strand

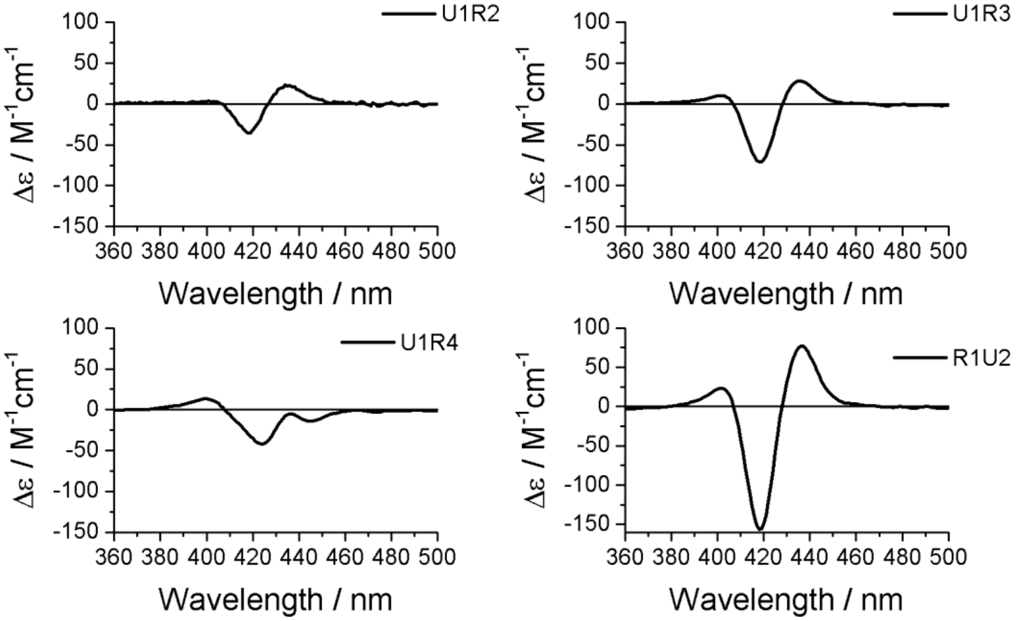

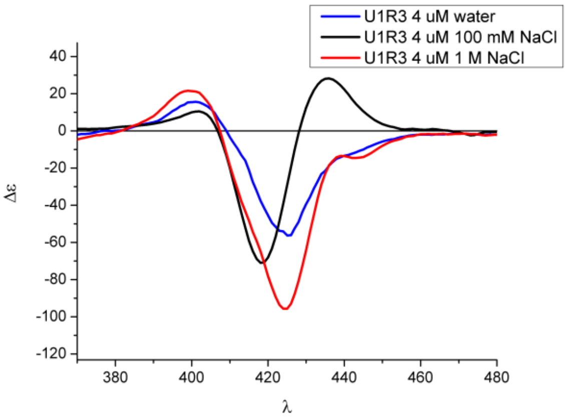

4) flexible linker with unmodified complementary strand

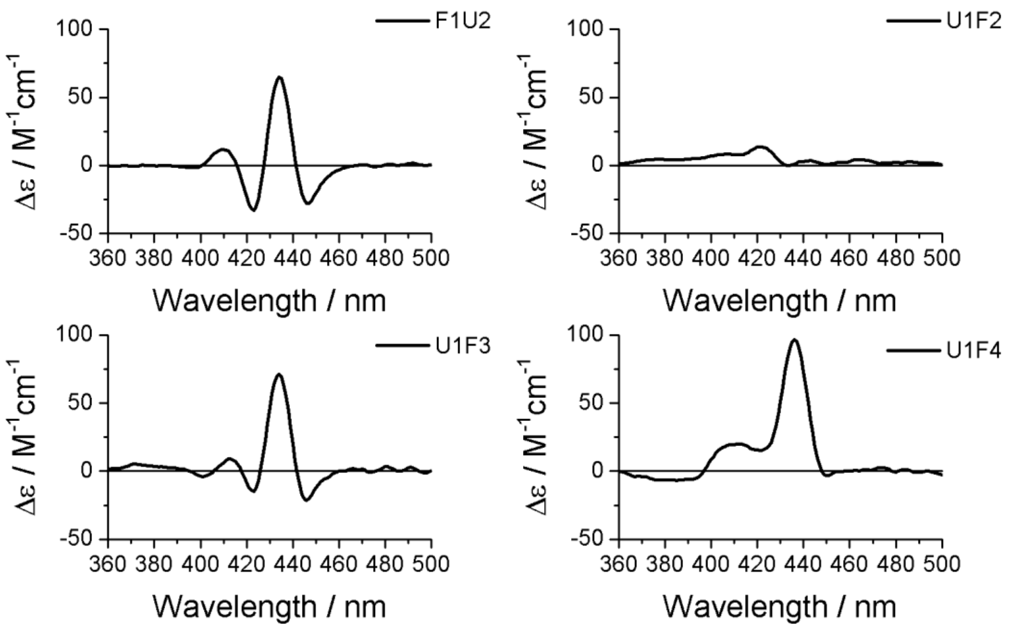

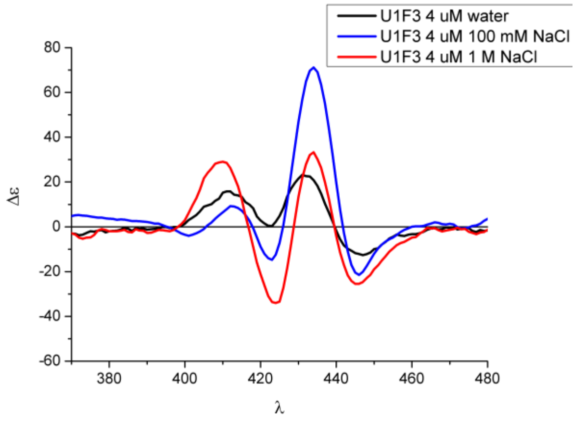

5) rigid linker, both strands modified with same porphyrin

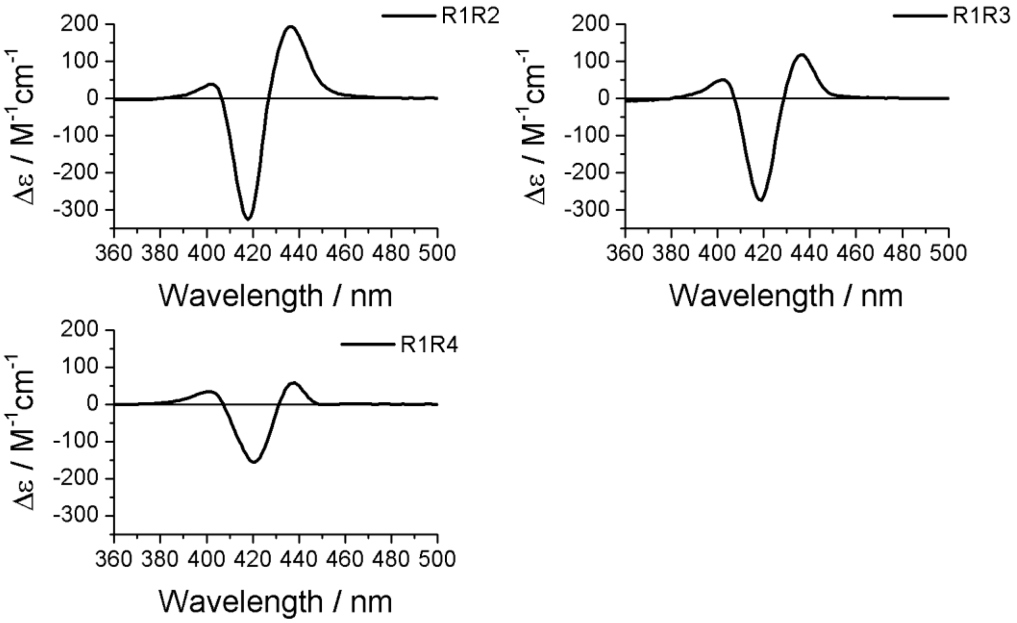

6) flexible linker, both strands modified with same porphyrin

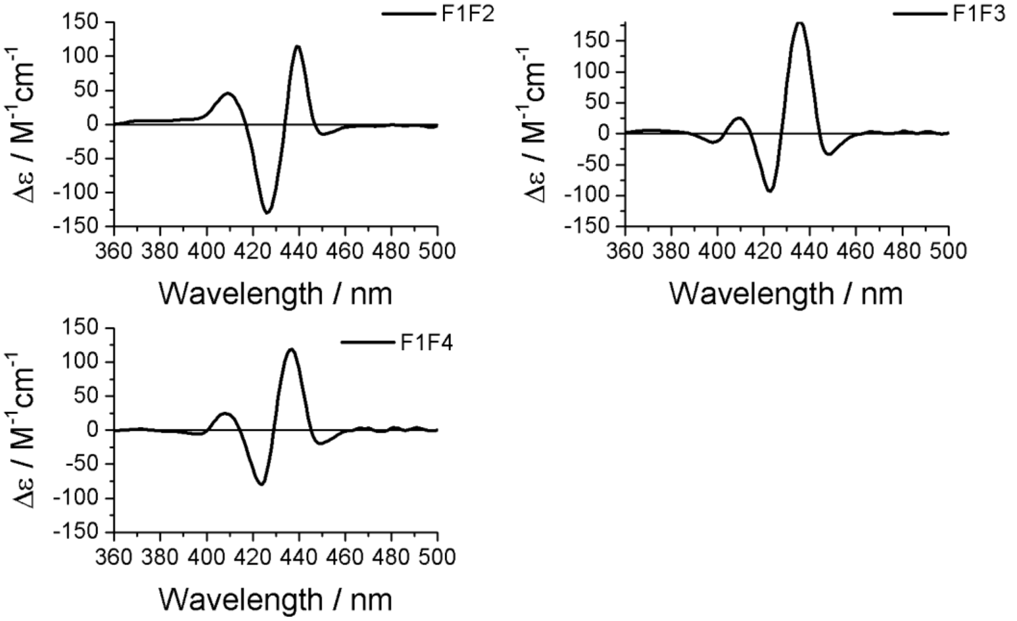

7) rigid/flexible linker mixed

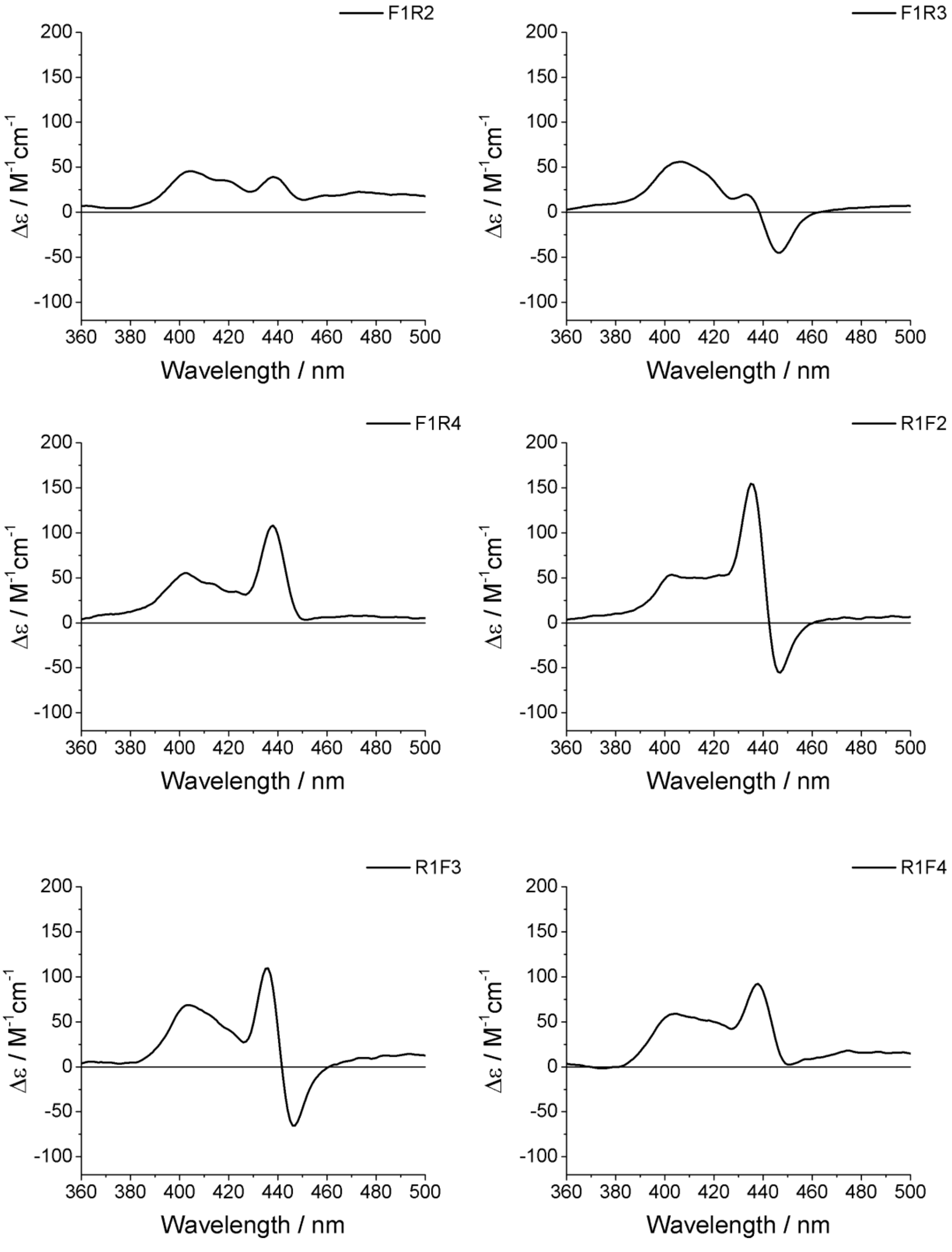

9) Zinc-metallated 2-porphyrin strand in duplex

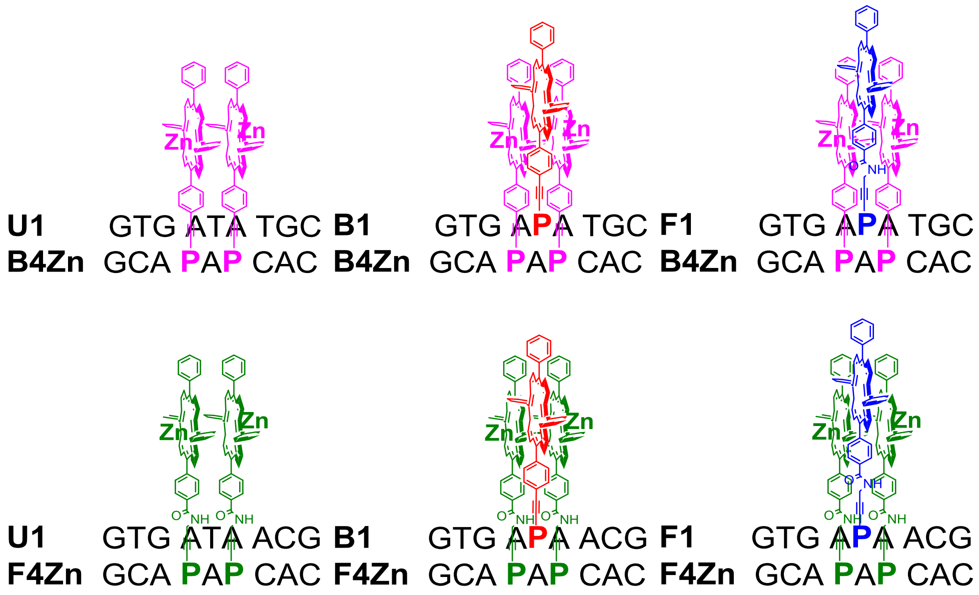

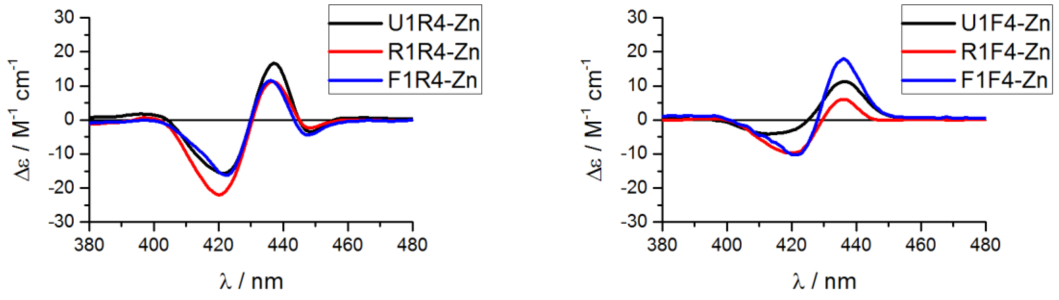

UV-vis / fluorescence spectra

Selected examples of ssDNA and dsDNA strands with the LNA building blocks incorporated.

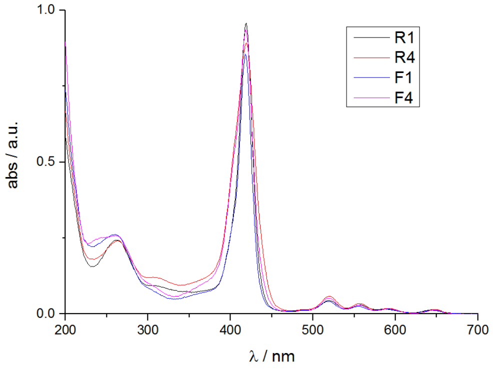

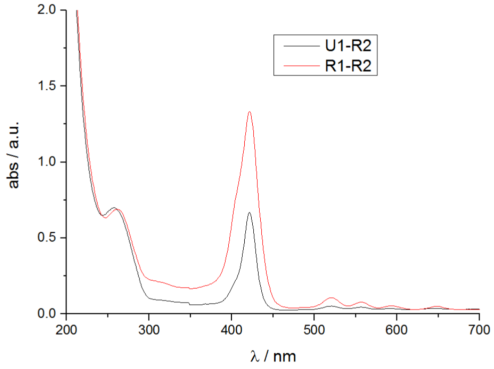

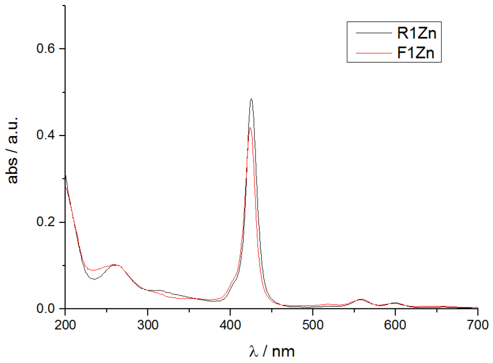

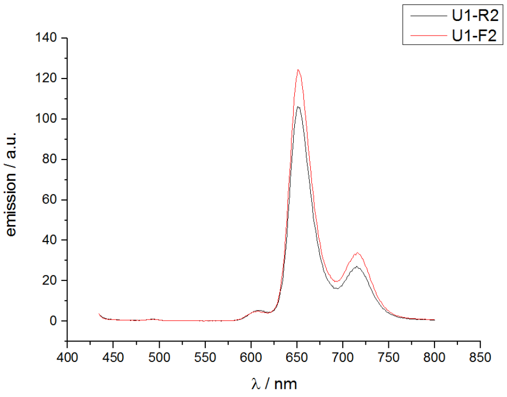

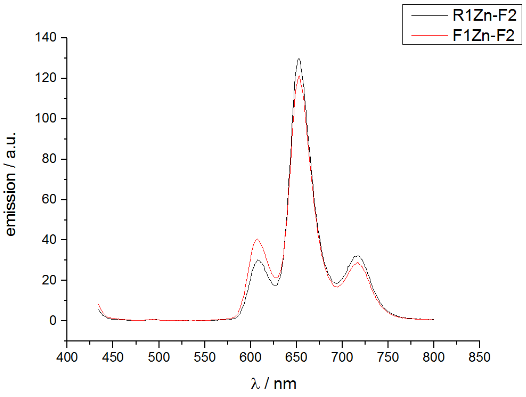

[1] I. Bouamaied, E. Stulz, *SYNLETT* **2004**, 1579-1583.
[2] L. A. Fendt, I. Bouamaied, S. Thöni, N. Amiot, E. Stulz, *J. Am. Chem. Soc.* **2007**, *129*, 15319-15329.
[3] P. Kumar, M. E. Østergaard, B. Baral, B. A. Anderson, D. C. Guenther, M. Kaura, D. J. Raible, P. K. Sharma, P. J. Hrdlicka, *J. Org. Chem.* **2014**, *79*, 5047-5061.
[4] A. Brewer, G. Siligardi, C. Neylon, E. Stulz, *Org. Biomol. Chem.* **2011**, *9*, 777-782.
[5] D. C. Guenther, P. Kumar, B. A. Anderson, P. J. Hrdlicka, *Chem. Commun.* **2014**, *50*, 9007-9009.
